# Highly Efficient and Stable Hydrogen Production in All pH Range by Two-Dimensional Structured Metal-Doped Tungsten Semicarbides

**DOI:** 10.34133/2019/4029516

**Published:** 2019-05-02

**Authors:** Edison H. Ang, Khang N. Dinh, Xiaoli Sun, Ying Huang, Jun Yang, Zhili Dong, Xiaochen Dong, Wei Huang, Zhiguo Wang, Hua Zhang, Qingyu Yan

**Affiliations:** ^1^Energy Research Institute, Interdisciplinary Graduate School, Nanyang Technological University, 637553, Singapore; ^2^Centre for Programmable Materials, School of Materials Science and Engineering, Nanyang Technological University, 639798, Singapore; ^3^Institute of Advanced Materials, Nanjing Tech University, Nanjing 210000, China; ^4^School of Physical Electronics, University of Electronic Science and Technology of China, Chengdu 610054, China; ^5^Department of Chemistry, City University of Hong Kong, Kowloon, Hong Kong

## Abstract

Transition-metal-doped tungsten semicarbide nanosheets (M-doped W_2_C NSs, M=Fe, Co, and Ni) have been synthesized through carburization of the mixture of tungsten trioxide, polyvinylpyrrolidone, and metal dopant. The nanosheets grow directly on the W mesh and have the lateral dimension of several hundreds of nm to a few *μ*m with a thickness of few tens nm. It is demonstrated that the M-doped W_2_C NSs exhibit superior electrocatalytic activity for hydrogen evolution reaction (HER). Impressively, the Ni-doped W_2_C NSs (2* at*% Ni) with the optimized HER activity show extremely low onset overpotentials of 4, 9, and 19 mV and modest Tafel slopes of 39, 51, and 87 mV dec^−1^ in acidic (pH=0), neutral (pH=7.2), and basic (pH=14) solutions, respectively, which is close to the commercial Pt/C catalyst. Density functional theory (DFT) calculations also demonstrate that the Gibbs free energy for H adsorption of Ni-W_2_C is much closer to the optimal value ∆G_H⁎_ = -0.073 eV as compared to -0.16 eV of W_2_C. Furthermore, nearly 100% Faradaic efficiency and long-term stability are obtained in those environments. This realization of highly tolerant metal semicarbide catalyst performing on par with commercial Pt/C in all range of pH offers a key step towards industrially electrochemical water splitting.

## 1. Introduction

Hydrogen generated by the electrolysis of water has become an increasingly attractive energy carrier due to its high energy density [1–4]. Electrocatalysts used for the hydrogen evolution reaction (HER) are important and key components for water splitting [5, 6]. It is well known that noble metals, such as Pt, are the most efficient HER electrocatalyst due to its fast reaction kinetics and low overpotential to drive the HER reaction [7, 8]. However, its high cost and low natural abundance hamper its wide applications [9–12]. Therefore, alternative electrocatalysts with low cost, good stability, and high catalytic activity are highly desirable.

In recent years, various nonnoble metal materials, such as phosphides [13, 14], sulfides [15–19], phosphosulfides [20], and carbides [21, 22], were prepared and tested as alternatives for Pt in the HER. Among the aforementioned materials, early transition metal carbides, especially tungsten carbides [23–25] with similar d-band electronic density-of-state to that of Pt, could be considered as effective nonnoble metal HER electrocatalysts [26, 27]. For the past decades, many efforts have been devoted to the synthesis of highly active tungsten carbides (WC) HER electrocatalyst because of the above-mentioned electronic structure and other unique properties, such as high electrical conductivity, high resistance to carbon monoxide and bisulfide poisoning, and excellent corrosion tolerance over the wide range of pH and potential [28]. Unfortunately, all reported tungsten carbides (WC) exhibited poor performance towards HER. Therefore, tungsten carbide has only been used as a catalyst support instead of carbon for Pt [24, 29].

On the other hand, tungsten semicarbide (W_2_C) is metal-terminated and theoretically predicted to have higher HER activity due to larger 5d-orbital electron population [30]. However, it has not been demonstrated experimentally, since the formation of W_2_C is not favorable below 1,250°C [31]. Even at high temperature, a mixture of WC and W_2_C phases often forms because the metastable W_2_C phase is readily transformed into stable WC phase in the presence of carbon [32]. Up to date, it has been reported that W_2_C possesses the onset overpotential of 50 mV, which is far higher than the Pt/C benchmark (~0 mV) [33]. It may be arisen from the lack of reliable synthetic approaches. High temperature reduction (usually > 1500°C) of W or W-containing precursors by gaseous carbon source results in coking of catalyst surface and uncontrollable sintering. Consequently, it leads to lower electrochemical active surface area and then poor catalytic performance. Moreover, the morphology control and catalytic tuning should also be taken into account. As known, two-dimensional (2D) nanostructures offer good electron transfer platform, superior electron mobility, high surface area, and more surface active sites, which has not been demonstrated for W_2_C up to date. Therefore, it is urgent to explore an appropriate method for selective synthesis of W_2_C with desired 2D nanostructures and tunable electrocatalytic properties toward HER.

Herein, we report a simple strategy to prepare metal-doped W_2_C nanosheets (NSs) on tungsten (W) substrates at a lower synthetic temperature (900°C) through the hydrothermal reactions followed by a carburization process. The M-doped W_2_C NSs (M=Fe, Co, and Ni) grown directly on the W mesh, with the lateral size of several hundreds of nm to a few *μ*m and the thickness of a few tens of nm, can be used as binder-free electrodes. This offers an efficient pathway for electron transport and the vertically aligned 2D nanostructure provides high surface area for HER. Among the M-doped W_2_C NSs, the Ni-doped W_2_C NSs (2* at*% Ni) electrocatalyst exhibits close-to-Pt HER performance with low onset overpotentials of 4, 9, and 19 mV and small Tafel slopes of 39, 51, and 87 mV dec^−1^ in acidic (pH=0), neutral (pH=7.2), and basic (pH=14) conditions, respectively. Moreover, it gives ~100% Faradaic yield and exhibits excellent stability towards the HER in those solutions. This outstanding performance can be attributed to its optimal |∆G_H*∗*_| value (close to zero) based on the density functional theory (DFT) calculations. This phase-pure W_2_C, with high electric conductivity, excellent tolerance, and the advantages of 2D nanostructure, would be of great interest to a wide range of research areas (*i.e.*, electrocatalysis, Li-O_2_ batteries, supercapacitor, and chemical and biological sensing), where electrical conductivity is one of the key parameters for high performance applications.

## 2. Results and Discussion

[Supplementary-material supplementary-material-1] (Supporting Information) illustrates the synthesis of M-doped W_2_C NSs. First, vertical growths of WO_3_ NSs were carried out on a W substrate by hydrothermal treatment of aqueous solution of Na_2_WO_4_·2H_2_O and NaCl (pH ~2) at 180°C (Step 1). The as-obtained WO_3_ NSs were then immersed into a mixture of aqueous MCl_2_ (MCl_2_=FeCl_2_, CoCl_2_, and NiCl_2_) dopant and polyvinylpyrrolidone (PVP) precursor, followed by heat treatment at 180°C to obtain the WO_3_/PVP/M mixture (Step 2). Finally, the as-prepared WO_3_/PVP/M mixture was carburized at 900°C under the H_2_/Ar environment to obtain the M-doped W_2_C NSs (Step 3).

**Figure 1:**
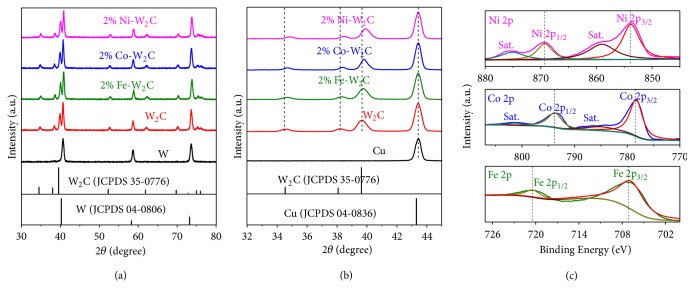
*XRD and XPS characterizations of various metal dopants in W*_*2*_ *C NSs*. (a) XRD patterns of W substrate, W_2_C, and 2% M-W_2_C NSs (M=Fe, Co, and Ni) on W substrate. (b) XRD patterns of Cu internal standard, W_2_C, and 2% M-W_2_C NSs (M=Fe, Co, and Ni) using Cu as internal standard. (c) High-resolution XPS spectra of Fe 2p, Co 2p, and Ni 2p for 2% M-W_2_C NSs (M=Fe, Co, and Ni).

**Figure 2:**
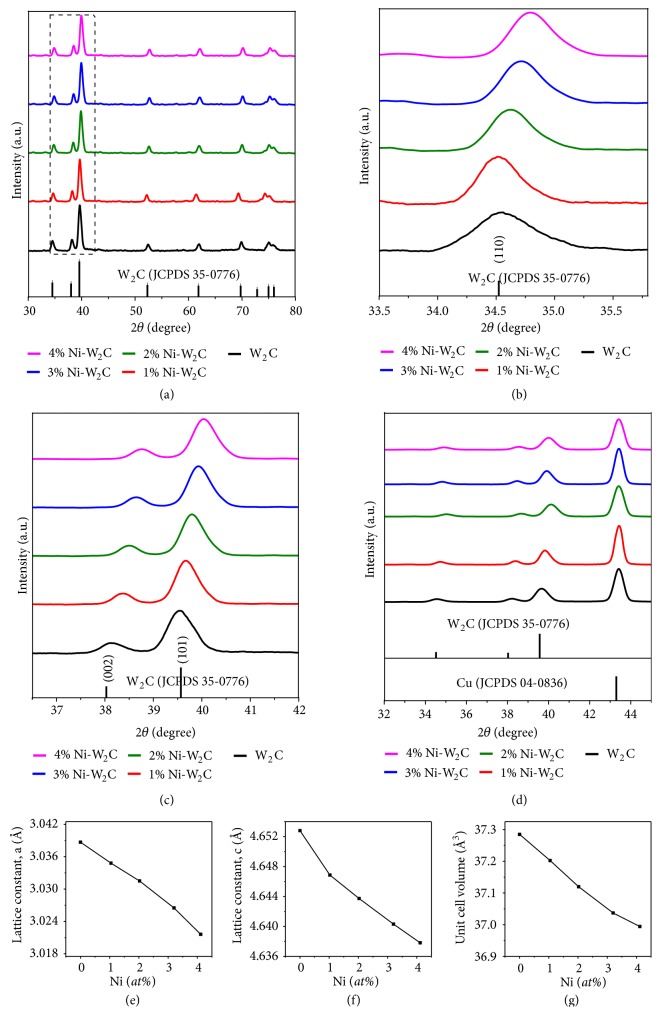
*XRD analyses of various doping contents in M-W*_*2*_ *C NSs*. (a) XRD patterns and (b, c) magnified XRD patterns of W_2_C and W_2_C with various Ni (*at*%) doping contents. (d) Magnified XRD patterns of W_2_C and W_2_C with various Ni (*at*%) doping contents using Cu as internal standard. (e, f) The plots of lattice parameters a and c versus Ni (*at*%) doping content measured by ICP-OES. (g) The plot of unit cell volume of W_2_C versus Ni (*at*%) doping content measured by ICP-OES.

In Figure 1(a), the X-ray diffraction (XRD) peaks located at 40.2, 58.2, and 73.2° correspond to the W substrate (JCPDS No. 04-0806). After growth of WO_3_ NSs on the W substrate, all the XRD peaks ([Supplementary-material supplementary-material-1], Supporting Information) can be indexed to the hexagonal WO_3_ (JCPDS No. 33-1387) and W (JCPDS No. 04-0806). After carburization, the XRD peaks (Figure 1(a)) match those of W_2_C (JCPDS No. 35-0776) with space group of P-3m1 (a = 0.30387 nm and c = 0.46528 nm) and W (JCPDS No. 04-0806). Moreover, M-doped W_2_C (M=Fe, Co, and Ni) samples with varied dopant content have also been prepared. The amount of metal dopants was determined by the inductively coupled plasma-optical emission spectroscopy (ICP-OES, [Supplementary-material supplementary-material-1], Supporting Information). Due to the difference in ionic sizes and ionic charges we can only dope up to 4* at*% of M into W_2_C lattice and 2* at*% M-doped W_2_C (namely, 2% M-W_2_C, M=Fe, Co, and Ni) was mainly used for detail characterizations. It is notable that the (110) peak of W (40.2°) overlaps with the (101) peak of W_2_C (39.6°). Therefore, to obtain accurate lattice constants for 2% M-W_2_C, the nanosheets were scrapped off from the W mesh and dropped cast onto Cu substrate; and the XRD peaks were calibrated with the crystalline Cu (JCPDS No. 04-0836) as an internal standard (Figure 1(b)). The XRD patterns of M-W_2_C (M=Fe, Co, and Ni) with varied doping content (0-4%) reveal that the diffraction peaks of (100), (002), and (101) at 34.5°, 38.0°, and 39.6°, respectively, slightly shift to the higher angles as compared to those of pure W_2_C (Figures 2(a)–2(d); Figures [Supplementary-material supplementary-material-1]–[Supplementary-material supplementary-material-1] and [Supplementary-material supplementary-material-1]–[Supplementary-material supplementary-material-1] in Supporting Information). It is worth noting that Cu as the internal reference did not show any detectable peak shift in the XRD measurements; hence, this kind of peak shift indicates the decrease of lattice parameters (*i.e.,* a and c as shown in [Supplementary-material supplementary-material-1] in Supporting Information) after M (M=Fe, Co, and Ni) was doped into the W_2_C lattice. To specify, the Rietveld refinement method [34] was performed to determine the changes of lattice parameters and the unit cell volumes with respect to the amount of dopant. The lattice parameters and consequently the unit cell volume decrease with the increased dopant content, implying that smaller Ni, Co, or Fe atoms have substituted for the W atoms randomly in the crystal structure (Figures 2(e)–2(g); Figures [Supplementary-material supplementary-material-1]–[Supplementary-material supplementary-material-1] and [Supplementary-material supplementary-material-1]–[Supplementary-material supplementary-material-1] in Supporting Information). Such observation is expected as the Ni, Co, and Fe atoms have smaller radii as compared to W [35].

The X-ray photon-electron spectroscopy (XPS) was also used to characterize the 2% M-W_2_C (M=Fe, Co, and Ni) (Figure 1(c)). The two strong peaks at 853.3 eV and 869.9 eV with two corresponding satellite peaks in the Ni 2p XPS spectrum can be assigned to the Ni^2+^ in Ni-C bond, which are the characteristic of Ni-doping in metal carbide materials [36, 37]. In the fine Co 2p XPS spectrum, peaks at binding energies of 778.4 eV and 793.4 eV and their satellites correspond to Co 2p_3/2_ and Co 2p_1/2_, indicating the presence of Co^2+^ and Co^3+^ in Co-C bond [37]. The peaks at 707.0 eV and 720.1 eV in the Fe 2p XPS spectrum are attributed to Fe^3+^ in Fe-C bond [37, 38]. All these results suggest that the Fe, Co, and Ni have been successfully doped into W_2_C.

**Figure 3:**
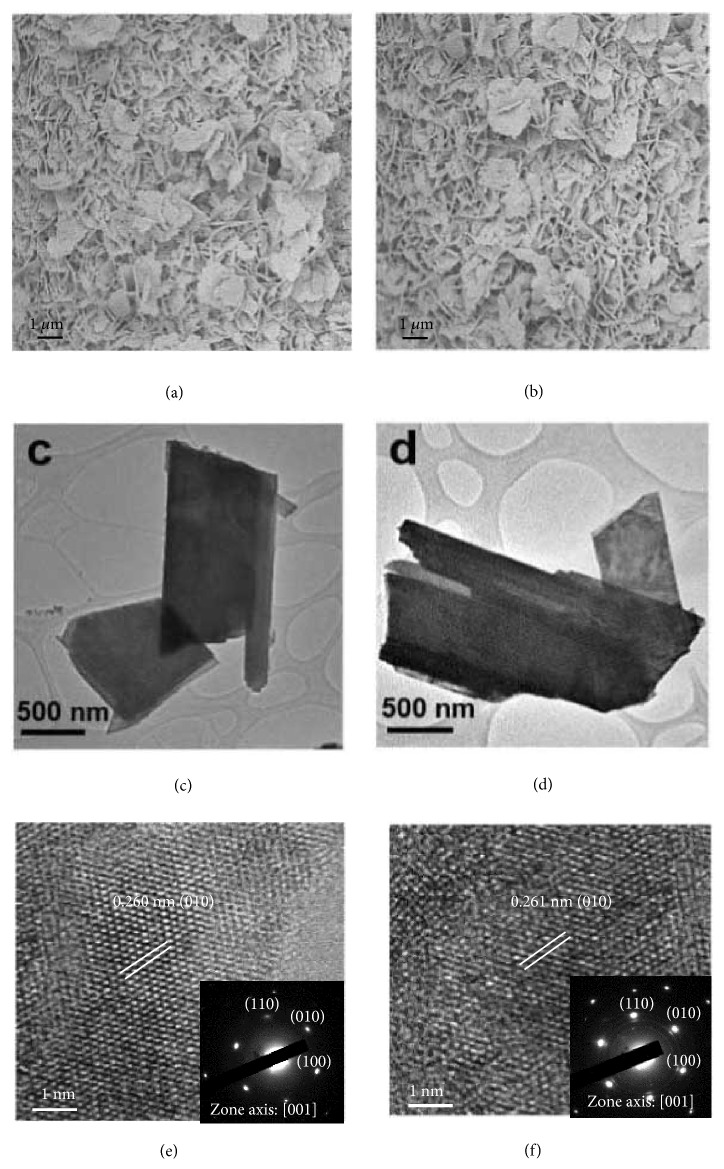
*SEM, TEM, and SAED measurements of W*_*2*_ *C and Ni-W*_*2*_ *C NSs*. (a, c, e) W_2_C and (b, d, f) 2% Ni-W_2_C NSs. (a, b) FESEM images. (c, d) Low-magnification TEM images. (e, f) HRTEM images (Insets: corresponding SAED patterns).

The scanning electron microscopy (SEM) images of W_2_C and 2% M-W_2_C (M=Fe, Co, and Ni) samples (Figures 3(a) and 3(b) and Figures [Supplementary-material supplementary-material-1] and [Supplementary-material supplementary-material-1] in Supporting Information) clearly show that the individual nanosheets were densely grown on the W mesh. The thickness of the whole nanosheet film on W mesh is ~1.0 *μ*m ([Supplementary-material supplementary-material-1], Supporting Information). The obtained W_2_C and 2% M-W_2_C nanosheets were then scraped off from the W mesh for the atomic force microscopy (AFM), transmission electron microscopy (TEM), and high-resolution (HR) TEM measurements. The AFM result confirmed that the thickness of the nanosheet is several tens of nm ([Supplementary-material supplementary-material-1], Supporting Information). As shown in the TEM images (Figures 3(c) and 3(d)), the shape of the NSs is irregular and the lateral dimension of the nanosheets is from several hundreds of nm to a few *μ*m. The HRTEM image of W_2_C nanosheet shows a lattice spacing of 0.260 nm (Figure 3(e)), corresponding to the d-spacing of (010) atomic planes of the W_2_C phase, whereas those lattice fringes for 2% M-W_2_C (M=Fe, Co, and Ni) are slightly higher at 0.261 nm (Figure 3(f) and Figures [Supplementary-material supplementary-material-1] and [Supplementary-material supplementary-material-1]), which is in a good agreement with the peak shift seen in XRD patterns. The selected area electron diffraction (SAED) patterns (insets in Figures 3(e) and 3(f) and Figures [Supplementary-material supplementary-material-1] and [Supplementary-material supplementary-material-1] in Supporting Information) show the single crystalline nature of the observed W_2_C and 2% M-W_2_C NSs (M=Fe, Co, and Ni) with exposure of (001) facets. The high-angle annular dark field (HAADF) images and scanning transmission electron microscopes-energy dispersive X-ray spectroscopy (STEM-EDX) mapping images ([Supplementary-material supplementary-material-1], Supporting Information) prove that W, C, and the doped metals are uniformly distributed over the 2% M-W_2_C NSs (M=Fe, Co, and Ni).

**Figure 4:**
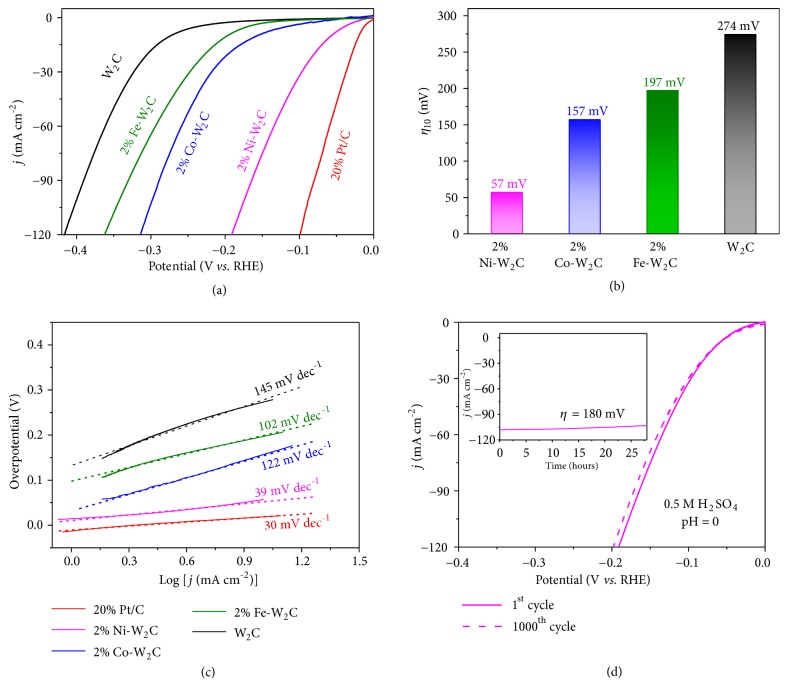
*HER electrochemical performances in acidic condition*. (a) Polarization curves of the 20% Pt/C, W_2_C, 2% Fe-W_2_C, 2% Co-W_2_C, and 2% Ni-W_2_C NSs at scan rate of 2 mV s^−1^ in 0.5 M H_2_SO_4_ solution (pH=0). (b) The overpotential of above catalysts at current density of 10 mA cm^−2^. (c) Corresponding Tafel plots. (d) Polarization curves of 2% Ni-W_2_C NSs before and after 1000 cyclic voltammetry cycles (Inset: chronoamperometry measurements at overpotential of 180 mV).

The HER electrocatalytic properties of M-W_2_C NSs (M=Fe, Co, and Ni) were studied using conventional 3-electrode setup in solutions with different pH values. Linear sweep voltammetry technique was performed at 2 mV s^−1^ to lower the capacitive current. All the measurements were carried out at room temperature (25°C) unless otherwise stated. For comparison, the W substrate, pure W_2_C NSs, and commercial Pt/C were also examined. We started the evaluations of the samples in H_2_-saturated 0.5 M H_2_SO_4_ (pH=0) solution (Figure 4). Firstly, it should be noted that the W substrate exhibits nearly negligible HER activity even at -0.3 V vs. RHE ([Supplementary-material supplementary-material-1], Supporting Information). For three types of doped samples (M-W_2_C, M=Ni, Co, and Fe), 2* at*% of metal doping leads to an optimal HER catalytic activity in all prepared samples ([Supplementary-material supplementary-material-1], Supporting Information). Compared to W substrate, the W_2_C nanosheets afford a much smaller onset overpotential, which could be further reduced by chemically doping metal M (M=Fe, Co, and Ni) into W_2_C lattice (Figure 4(a)). As summarized in [Supplementary-material supplementary-material-1] in Supporting Information, the pure W_2_C, 2% Fe-W_2_C, 2% Co-W_2_C, and 2% Ni-W_2_C NSs electrocatalysts exhibit onset overpotentials of 122, 78, 45, and 4 mV, respectively, in 0.5 M H_2_SO_4_ solution (pH=0). In addition, the operating overpotentials required to drive a cathodic current density of 10 mA cm^−2^ (*η*_10_) are 274, 197, 157, and 57 mV for pure W_2_C, 2% Fe-W_2_C, 2% Co-W_2_C, and 2% Ni-W_2_C NSs, respectively (Figure 4(b)). Clearly, the 2% Ni-W_2_C electrocatalyst demonstrates the lowest onset overpotential and *η*_10_ as compared to other control samples and approaches close to Pt (~0 onset overpotential and *η*_10_ of 23 mV). The Tafel slopes are 145, 102, 122, and 39 mV dec^−1^ for the pure W_2_C, 2% Fe-W_2_C, 2% Co-W_2_C, and 2% Ni-W_2_C NSs, respectively (Figure 4(c)). It means that the HER for pure W_2_C, 2% Fe-W_2_C, and 2% Co-W_2_C proceeds through the Volmer-Heyrovsky mechanism, in which the Volmer reaction is the rate-limiting step [39], whereas the HER for 2% Ni-W_2_C NSs follows the Volmer-Tafel reaction process, in which the recombination of adsorbed hydrogen atoms is the rate-determining step [39]. Notably, the Tafel slope of 2% Ni-W_2_C NSs is close to the commercial 20% Pt/C electrocatalyst (30 mV dec^−1^), suggesting that 2% Ni-W_2_C NS electrode might be used to replace the expensive Pt electrocatalyst for HER. The inherent activities toward HER were also evaluated by the exchange current density. The 2% Ni-W_2_C still performs well at 0.79 mA cm^−2^, which is far higher than W_2_C (0.19 mA cm^−2^), 2% Fe-W_2_C (0.22 mA cm^−2^), and 2% Co-W_2_C (0.41 mA cm^−2^) and is just slightly below Pt/C (0.92 mA cm^−2^).

**Figure 5:**
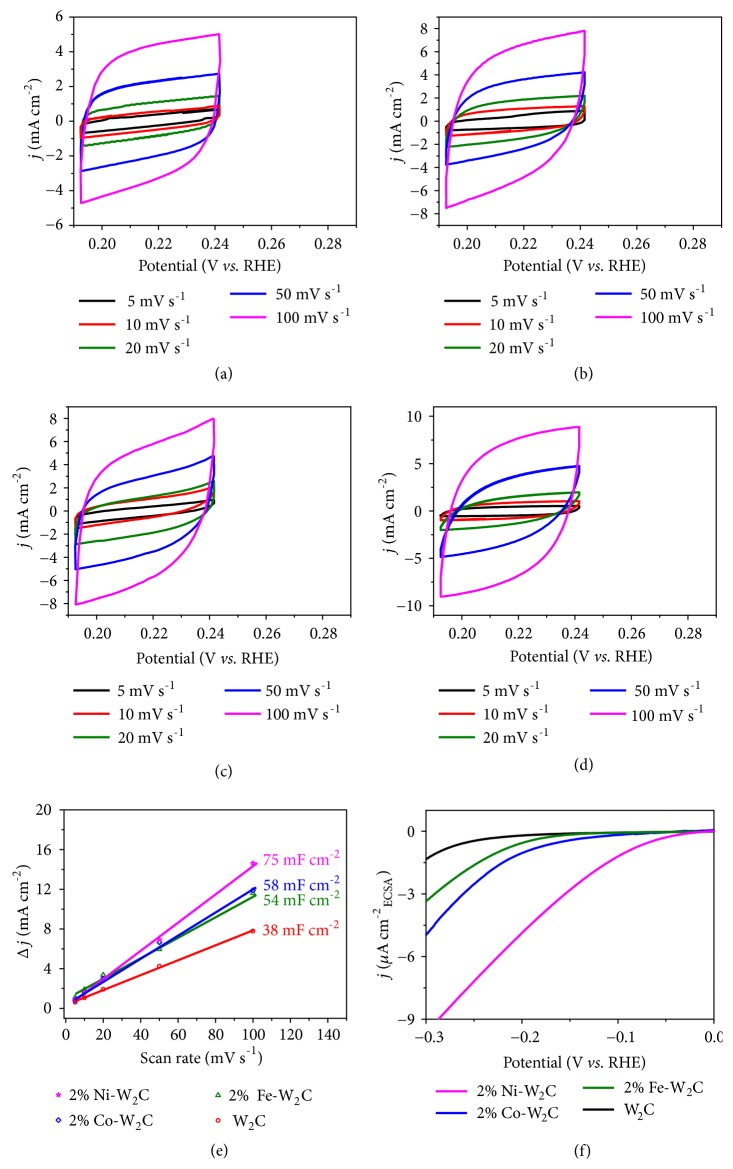
*ECSA analysis*. Cyclic voltammograms (CVs) of (a) W_2_C, (b) 2% Fe-W_2_C, (c) 2% Co-W_2_C, and (d) 2% Ni-W_2_C NSs obtained in a potential window of 0.192-0.242 V (*vs.* RHE) at various scan rates of 5, 10, 20, 50, and 100 mV s^−1^ in 0.5 M H_2_SO_4_ solution. (e) The capacitive current (j_anodic_-j_cathodic_) at 0.22 V (*vs.* RHE) as a function of the scan rate for the W_2_C and 2% M-W_2_C (M=Fe, Co, and Ni) NSs. (f) HER polarization curves of W_2_C, 2% Fe-W_2_C, 2% Co-W_2_C, and 2% Ni-W_2_C NSs after electrochemical active area (ECSA) normalization.

In light of the high electrocatalytic activity of 2% M-W_2_C NSs (M=Fe, Co, and Ni), the electrochemical effective surface area (ESCA), which is proportional to the measured double-layer capacitance (C_dl_), was determined using cyclic voltammetry (Figures 5(a)–5(d)). The C_dl_ values of the W_2_C, 2% Fe-W_2_C, 2% Co-W_2_C, and 2% Ni-W_2_C electrodes are 38, 54, 58, and 75 mF cm^−2^, respectively (Figure 5(e)). The 2- to 2.5-fold higher ESCA of 2% M-W_2_C NSs as compared to the pure W_2_C indicates that the number of surface active sites significantly increased after the substitutional doping of transition metal atom (*e.g.,* Fe, Co, or Ni) in W_2_C NSs. Importantly, after ECSA normalization, the HER activity of 2% Ni-W_2_C NSs is still the best (Figure 5(f)). Hence, the enhancement seen in HER activity is attributed not only to the increase of ECSA but also to the high intrinsic activity of 2% M-W_2_C NSs, especially 2% Ni-W_2_C NSs. Electrochemical impedance spectroscopy (EIS) results ([Supplementary-material supplementary-material-1], Supporting Information) compare the charge transfer resistance (R_ct_) of pure W_2_C and 2% M-W_2_C (M=Fe, Co, and Ni) electrodes. The obtained R_ct_ values of pure W_2_C, 2% Fe-W_2_C, 2% Co-W_2_C, and 2% Ni-W_2_C are 43.8 Ω, 29.0 Ω, 25.7 Ω, and 12.6 Ω, respectively. The lowest R_ct_ of 2% Ni-W_2_C could be attributed to the fast reaction rate for the proton reduction on the electrocatalyst surface.

Due to the best performance of 2% Ni-W_2_C in acidic condition, it is necessary to evaluate its long-term durability. Continuous CV was performed between 0.2 and -0.3 V (*vs.* RHE) at a scan rate of 100 mV s^−1^ in 0.5 M H_2_SO_4_ solution (Figure 4(d)). As can be seen, the polarization curves before and after 1000 CV cycles almost overlap with each other. Chronoamperometry measurement of 2% Ni-W_2_C NSs at overpotential of 180 mV also shows a stable current density of 108 mA cm^−2^ for 28 hours (Figure 4(d), inset). The post HER analysis,* i.e.*, XRD, XPS, and SEM (Figures [Supplementary-material supplementary-material-1], [Supplementary-material supplementary-material-1], [Supplementary-material supplementary-material-1], and [Supplementary-material supplementary-material-1] in Supporting Information), shows almost no change observed, revealing high structural and chemical stability. All these results suggest the remarkable stability and durability of the synthesized 2% Ni-W_2_C NSs in such HER process.

**Figure 6:**
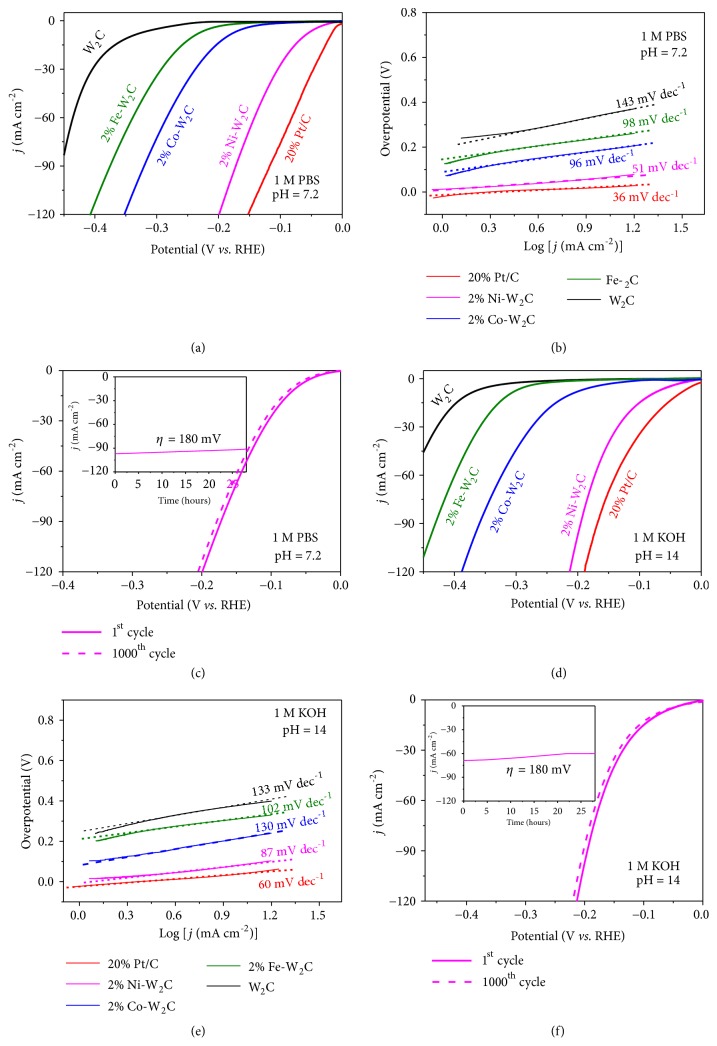
*HER electrochemical performances in neutral and alkaline condition*. (a-c) 1 M PBS (pH=7.2) and (d-f) 1 M KOH (pH=14) solutions. (a, d) Polarization curves of the 20% Pt/C, W_2_C, 2% Fe-W_2_C, 2% Co-W_2_C, and 2% Ni-W_2_C NSs and their corresponding (b, e) Tafel plots. (c, f) Polarization curves of 2% Ni-W_2_C NSs before and after 1000 cyclic voltammetry cycles (Inset: chronoamperometry measurements at overpotential of 180 mV).

An ideal HER electrocatalyst should not only have comparable activity/efficiency to Pt/C in 0.5 M H_2_SO_4_, but also acquire high catalytic activity and good stability over a wide pH range. Therefore, we further examine the electrochemical performance of the 2% M-W_2_C NSs (M=Fe, Co, and Ni) in neutral (1 M phosphate buffer, pH=7.2) and basic (1 M KOH, pH=14) solutions. In neutral condition, the reductive sweep of W_2_C reveals a high *η*_10_ of 334 mV for HER (Figure 6(a)). In contrast, noticeable enhancement was obtained with lower *η*_10_ (242 mV, 188 mV, and 63 mV for 2% Fe-W_2_C, 2% Co-W_2_C, and 2% Ni-W_2_C, respectively) and sharply increased cathodic current. Interestingly, the 2% Ni-W_2_C still displays favorable performance, which is further shown by its modest onset overpotential and Tafel slope of 9 mV and 51 mV dec^−1^, respectively (Figure 6(b)), while the values for W_2_C, 2% Co-W_2_C, and 2% Fe-W_2_C are less attractive at 227 mV and 143 mV dec^−1^; 67 mV and 96 mV dec^−1^; 123 mV and 98 mV dec^−1^, respectively. Similarly, HER catalytic activity in basic condition is presented in Figures 6(d) and 6(e). The onset overpotential and *η*_10_ for 2% Ni-W_2_C are 19 mV and 81 mV, respectively, surpassing the 2% Co-W_2_C (85 mV and 213 mV), 2% Fe-W_2_C (188 mV and 312 mV), and W_2_C (226 mV and 380 mV) by a great margin. The Tafel slope for 2% Ni-W_2_C in KOH solution is 87 mV dec^−1^, which is slightly worse than that of the commercial 20% Pt/C (60 mV dec^−1^) and is much lower than those of 2% Co-W_2_C (130 mV dec^−1^), 2% Fe-W_2_C (102 mV dec^−1^), and W_2_C (133 mV dec^−1^). Furthermore, these values of 2% Ni-W_2_C are much better than the reported electrocatalysts (Tables [Supplementary-material supplementary-material-1]–[Supplementary-material supplementary-material-1], Supporting Information).

The stability and durability of 2% Ni-W_2_C in PBS and KOH solution were also investigated by continuous CV and chronoamperometry method (Figures 6(c) and 6(f)). Less than 5% changes in current density are observed within 28 hours of electrolysis at 180 mV overpotential in both solutions. After 1000 CV scans, the reductive sweep voltammetry shows a slight negative shift compared to the initial one (Figures 6(c)–6(f), inset). In addition, the SEM results for 2% Ni-W_2_C after the durability test indicate no obvious changes in the 2D morphology (Figures [Supplementary-material supplementary-material-1] and [Supplementary-material supplementary-material-1], Supporting Information). Similarly, the XRD patterns (Figures [Supplementary-material supplementary-material-1] and [Supplementary-material supplementary-material-1], Supporting Information) of the 2% Ni-W_2_C NSs samples after chronoamperometry measurements for 28 h, specifically the diffraction peaks at 34.5°, 38.0°, and 39.6° corresponding to (100), (002), and (101) planes, respectively, resemble those of the W_2_C (JCPDS 35-0776) in acidic, neutral, and alkaline solutions. These detectable peaks indicate that the phases of the samples remain unchanged after long-term HER testing. Equally important, Figures [Supplementary-material supplementary-material-1] and [Supplementary-material supplementary-material-1] in Supporting Information show the binding energies of the 2% Ni-W_2_C NSs samples at 853.3 eV (Ni 2p_3/2_) and 869.9 eV (Ni 2p_1/2_) which are attributed to the Ni dopant in the W_2_C phase. These noticeable peaks imply that the chemical structures of Ni dopant in the W_2_C structure remain unchanged after durability test for 28 h in various pH solutions. On top of that, quantitative XPS analyses show almost no nickel leaching ([Supplementary-material supplementary-material-1], Supporting Information). Those results demonstrate that the 2% Ni-W_2_C possesses remarkable stability in HER under neutral and basic condition, suggesting the promise for implementing this new catalyst into realistic cathodic electrode for water splitting.

Faradaic efficiency tests in the pH solutions of 0, 7.2, and 14 were also conducted ([Supplementary-material supplementary-material-1], Supporting Information). For experimental amount of H_2_ generated, headspace samples were taken for gas chromatography every 20 minutes while operating continuously at -80 mA cm^−2^. The theoretical volume of H_2_ evolved was calculated by Faraday's law with the assumption that all electrons passing through the circuit engage in proton reduction. The experimental and theoretical amounts of H_2_ generated are in a good agreement, showing almost 100% current to hydrogen productivity.

**Figure 7:**
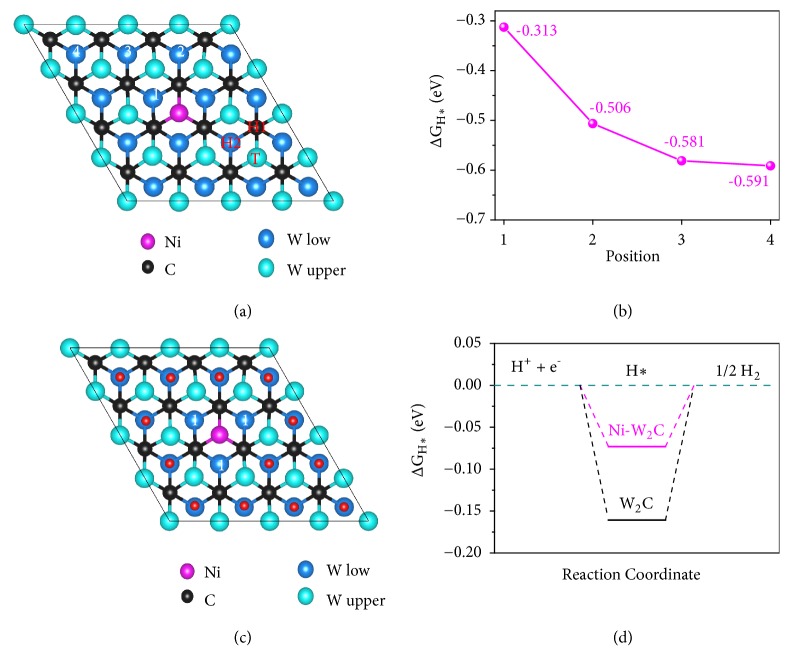
*DFT calculations*. (a) Atomistic configuration of Ni-W_2_C nanosheet (T, H1, and H2 are the possible adsorption sites for H on W_2_C nanosheet; 1-4 are the possible adsorption sites for H on Ni-W_2_C nanosheet). (b) Gibbs free-energies for H adsorbed at sites 1-4 on Ni-W_2_C nanosheet. (c) Atomistic configuration of Ni-W_2_C nanosheet at high hydrogen adsorption coverage. (d) Free-energy diagrams for HER of W_2_C and Ni-W_2_C high hydrogen adsorption coverage.

To understand the effect of the Ni dopant in W_2_C toward the HER activity, a systematic calculation on the electronic properties of pure W_2_C and Ni-W_2_C was carried out by employing DFT calculations (details of simulation method can be seen in the experimental section in Supporting Information). The proposed surface active sites of the Ni-W_2_C were then theoretically predicted by the HER free energy diagrams. The overall HER pathway can be described by a three-state diagram: (1) an initial state (H^+^ + e^−^), (2) an intermediate state (adsorbed H*∗*), and (3) a final state (1/2 H_2_ product) [7]. As known, the optimal value of Gibbs free energy of H*∗* adsorption, |∆G_H*∗*_|, should be zero, leading to the optimal HER electrocatalytic activity. Negative ∆G_H*∗*_ implies that the desorption of H*∗* is to be the rate-determining step (RDS), while positive ∆G_H*∗*_ means that the formation of intermediate H*∗* is the RDS [7]. As shown in Figure 7(a), there are three possible adsorption sites for hydrogen on W_2_C nanosheet,* i.e.*, the top of W atom (T), two trigonal sites with superimposing with C (H1), and bottom W atoms (H2). Based on the calculations, H prefers to be adsorbed at the H2 sites with lowest free energy of -0.71 eV ([Supplementary-material supplementary-material-1], Supporting Information). With Ni doping, we firstly investigated the energy-preferable adsorption site out of 4 H2 sites in the configuration (sites 1-4 in Figure 7(a)). As shown in Figure 7(b), the H adsorbed on site 4, which is far away from the doping position, has the lowest free energy. This reveals that the H will be preferable to be adsorbed firstly on the sites away from the doping position. Therefore, at high hydrogen adsorption coverage, the sites far away from the doping position are preferably occupied by hydrogen (Figure 7(c)). In this case, the calculated |∆G_H*∗*_| value is 0.073 eV, whereas the pristine W_2_C shows a much higher |∆G_H*∗*_| value of 0.16 eV at high coverage (Figure 7(d)). These results indicate that the Ni incorporation would significantly enhance the hydrogen adsorption/desorption process, and thus, catalytic activity of W_2_C towards HER, which is in a good agreement with the experimental data.

## 3. Conclusion

In summary, we have successfully synthesized M-W_2_C NSs (M=Fe, Co, and Ni) on W meshes. The M-W_2_C NSs electrocatalysts show remarkable HER activities. Particularly the 2% Ni-W_2_C NSs exhibit low onset overpotentials of 4 mV, 9 mV, and 19 mV alongside with modest Tafel slopes of 39 mV dec^−1^, 51 mV dec^−1^, and 87 mV dec^−1^ in acidic (0.5 M H_2_SO_4_, pH=0), neutral (1 M PBS, pH=7.2), and basic (1 M KOH, pH=14) solutions, respectively. Importantly, the 2% Ni-W_2_C exhibits excellent Faradaic efficiency and long-cycling stability in those environments. DFT calculations further confirm the effectiveness of Ni incorporation by reducing |∆G_H*∗*_| significantly from 0.16 eV of W_2_C to 0.073 eV of Ni-W_2_C. This realization of nonnoble metal binder-free electrode with high tolerance and close-to-Pt electrocatalytic activity in a wide range of pH makes 2% Ni-W_2_C a promising contender for future development of H_2_ generation via electrochemical water splitting.

## 4. Materials and Methods

### 4.1. Growth of WO_*3*_ NSs on W Mesh

Firstly, the WO_3_ NSs were grown on the tungsten substrate using the hydrothermal method. Tungsten substrate was cleaned by sonication in a mixture of deionized water and ethanol (1:1 v/v ratio) for 15 min followed by drying at 50°C in vacuum oven. In a typical process, 0.4 mmol of Na_2_WO_4_.2H_2_O (Sigma-Aldrich) and 3.4 mmol of NaCl (Sigma-Aldrich) were dissolved in 6 mL of deionized water. Then 4.0 M of HCl (Merck, USA) aqueous solution was added dropwise to adjust the pH to 2.0. The solution obtained was transferred to a 23-mL Teflon-lined stainless-steel autoclave where the reaction was maintained at 180°C for 5 h. The synthesized WO_3_ NSs electrode was then washed sequentially with deionized water and ethanol and then dried in an oven at 50°C.

### 4.2. Synthesis of M-W_*2*_C NSs

In a typical synthetic procedure of W_2_C NSs, a mass ratio of polyvinylpyrrolidone (PVP, average mol wt 40,000) to WO_3_ NSs (40:1) was homogeneously mixed in 4 mL of deionized water. For M-W_2_C synthesis, various M dopants (*i.e.*, FeCl_2_, CoCl_2_, and NiCl_2_, Sigma-Aldrich) were also added to the solution mixture. The amount of M dopants in M_x_W_2-x_C samples was 0, 1, 2, 3, and 4* at*%, where x = 0, 0.03, 0.06, 0.09, and 0.12, respectively. Then, the solution mixture containing the as-synthesized WO_3_ NSs, PVP, and M-dopants precursors was transferred to a 23-mL Teflon-lined stainless-steel autoclave. The reaction was maintained at 180°C for 8 h. After the reaction was completed, the obtained WO_3_/PVP/M hybrids were then washed with deionized water and ethanol and dried at 50°C in an oven. Finally, the as-prepared WO_3_/PVP/M hybrids were put in the quartz boat and calcined at 900°C for 30 min under H_2_/Ar flow (V_H2_/V_Ar_=5/95, 300 mL min^−1^) at a ramping rate of 10°C min^−1^. The final product (M-W_2_C NSs) was washed with ethanol several times before it was collected for further characterizations.

### 4.3. Materials Characterization

X-ray diffraction was performed to characterize the sample on the Shimadzu XRD-6000 X-ray diffractometer with Cu-K*α* irradiation (*λ* = 1.5406 Angstrom). The morphology and structure of the materials were characterized using transmission electron microscopy with scanning transmission electron microscopy (JEOL, Model JEM-2100F, 2010 UHR; JEM-ARM200F) operating at 200 keV, field emission scanning electron microscopy (FESEM, JEOL, JSM-7600F), and atomic force microscopy (AFM) (Digital Instruments). The energy-dispersive X-ray spectroscopy (EDX), elemental mapping, and high angle annular dark field scanning transmission electron microscopy (HAADF-STEM) were performed by TEM (JEOL JEM 2100, 200 kV). The amounts of various M dopants and W contents in the M_x_W_2-x_C samples were determined using Dual-view Optima 5300 DV ICP-OES. Rietveld refinement method was processed using the TOPAS software. The X-ray photoelectron spectroscopy (XPS, Kratos AXIS Supra) spectra were conducted using Al anode.

### 4.4. Electrochemical Measurement

Electrochemical measurements were performed in a conventional three-electrode system using graphite rod as the counter electrode and the as-synthesized M-W_2_C, W_2_C NSs on W substrate as working electrode. Saturated calomel electrode served as the reference electrode in acidic and neutral solutions while Hg/HgO served as the reference electrode in basic solution. For comparison, 20% Pt/C catalyst slurry in IPA/Nafion mixture (0.95/0.05 v/v ratio) was drop-casted on W substrate and used as working electrode. Doctor blade was also employed to make sure the catalyst loadings are at 1 mg cm^−2^. All measurements were carried out in H_2_-purged 0.5 M H_2_SO_4_ (pH=0), 1 M phosphate buffer (pH=7.2), and 1 M KOH (pH=14) electrolytes. For the linear sweep voltammetry (LSV) measurements, the scan rates were set to be 2 mV s^−1^ to minimize the capacitive current. All the potentials were calibrated to a reversible hydrogen electrode (RHE) by using the following equations:

(1) $$\begin{array}{c} E_{RHE}=E_{SCE}+0.059\;\;x\;\;pH+{E_{SCE}}^{o}\;\left(\text{where }{E_{SCE}}^{o}=0.241\;V\right).\\ E_{RHE}=E_{Hg/HgO}+0.059\;\;x\;\;pH+{E_{Hg/HgO}}^{o}\;\left(\text{where }{E_{Hg/HgO}}^{o}=0.098\;V\right). \end{array}$$

All HER results were corrected for all ohmic (IR) losses throughout the system. To obtain the ohmic resistance, the electrochemical impedance spectroscopy (EIS) measurements were performed with frequency from 0.1 Hz to 100 kHz at an amplitude of 10 mV. The electrochemical surface area (ESCA) was estimated from the double-layer capacitance (C_dl_) of the films. The C_dl_ was determined with a simple cyclic voltammetry (CV) method. The CV was conducted in a potential window (0.192-0.242 V vs. RHE) at various scan rates of 5, 10, 20, 50, and 100 mV s^−1^. Then capacitive current (j_anodic_ - j_cathodic_) at 0.22 V vs RHE was plotted against various scan rates, while the slope obtained was divided by two to obtain the C_dl_ value. The Faradaic efficiency of the catalysts was determined by passing 80 mA cm^−2^ of current density through the water electrolysis system and the hydrogen gas generated was determined by analyzing 500 *μ*l of headspace samples via gas chromatography. The Faradaic efficiency is then defined as the ratio of the measured amount of H_2_ to that of the theoretical amount of H_2_ (based on Faraday's law).

### 4.5. Simulation Details and Methods

All the calculations were performed by using density functional theory (DFT) as implemented in the Vienna* ab initio* package (VASP) [40]. The projector augmented wave (PAW) method [41] was used to describe electron-ion interaction, while the generalized gradient approximation using the Perdew-Burke-Ernzerhof (PBE) functional was used to describe the electron exchange-correlation. A plane wave basis was set up to an energy cutoff of 520 eV. A 4 × 4 supercell of W_2_C monolayer was used to investigate the adsorption of hydrogen. A 30 Å vacuum space was constructed to avoid the periodical image interactions between periodical interactions. The Brillouin zone was integrated using the Monkhorst-Pack scheme [42] with 3 × 3 × 1* k*-grid. All the atomic positions and cell parameters were relaxed using a conjugate gradient minimization until the force on each atom is less than 0.01 eV Å^−1^.

Gibbs free-energy of the H adsorption was calculated using equation (2):

(2) $$\begin{array}{c} ∆G_{H}=∆E_{H}+∆E_{ZPE}-T∆S_{H} \end{array}$$

where ∆*E*_ZPE_ and ∆*S*_H_ are the zero-point energy and entropy difference of hydrogen in the adsorbed state and the gas phase, respectively. The hydrogen adsorption energy ∆*E*_H_ is calculated by the following expression:

(3) $$\begin{array}{c} ∆E_{H}=E_{Ni+nH}-E_{Ni+\left(n-1\right)H}-\frac{1}{2}E_{H_{2}} \end{array}$$

where *E*_*Ni*+*nH*_ and *E*_*Ni*+(*n*−1)*H*_ are the total energy of Ni-W_2_C nanosheet with* n*-th and (n-1)-th H atoms adsorption, respectively. *E*_H_2__ is the energy of a gas-phase hydrogen molecule.

The calculated frequency of H_2_ gas is 4345 cm^−1^. The contribution from the configurational entropy in the adsorbed state is small and neglected. So the entropy of hydrogen adsorption as △*S*_*H*_ = (1/2)*S*_*H*2_ where *S*_*H*_2__ is the entropy of molecule hydrogen in the gas phase at standard conditions. With these values, the Gibbs free energy from equation (2) can be rewritten as

(4) $$\begin{array}{c} ∆G_{H}=∆E_{H}+0.29 \end{array}$$

## Data Availability

All data generated or analyzed during this study are included in this published article and its Supplementary Materials.

## Supplementary Materials

Supplementary Materials Scheme 1: schematic diagram illustrating the growth process of M-W_2_C NSs (M=Fe, Co, and Ni) on W substrate. Step 1: growth of WO_3_ NSs on the W substrate. Step 2: growth of the WO_3_/PVP/M on the presynthesized WO_3_ NSs. Step 3: formation of M-W_2_C NSs by carburizing the WO_3_/PVP/M NSs for HER. Figure S1: XRD pattern of WO_3_ NSs on W substrate. Figure S2: (a) XRD patterns, (b, c) magnified XRD patterns of W_2_C and W_2_C with various Fe (*at*%) doping contents, (d) magnified XRD patterns of W_2_C and W_2_C with various Fe (*at*%) doping contents using Cu as internal standard, (e, f) the plots of lattice parameters a and c versus Fe (*at*%) doping content measured by ICP-OES, and (g) the plot of unit cell volume of W_2_C versus the Fe (*at*%) doping content measured by ICP-OES. Figure S3: (a) XRD patterns, (b, c) magnified XRD patterns of W_2_C and W_2_C with various Co (*at*%) doping contents, (d) magnified XRD patterns of W_2_C and W_2_C with various Co (*at*%) doping contents using Cu as internal standard, (e, f) the plots of lattice parameters a and c versus Co (*at*%) doping content measured by ICP-OES, and (g) the plot of unit cell volume of W_2_C versus Co (*at*%) doping content measured by ICP-OES. Figure S4: schematic representation of the crystal structure of hexagonal W_2_C with a space group of P-3m1. Figure S5: SEM and TEM characterizations of (a, c) 2% Fe-W_2_C and (b, d) 2% Co-W_2_C NSs, (a, b) FESEM images, and (c, d) HRTEM images (insets: corresponding SAED patterns). Figure S6: SEM image of the cross-section view of pure W_2_C NSs on W substrate. Figure S7: AFM image of pure W_2_C NSs and the corresponding height profile along the white dashed line. Figure S8: HAADF images and their corresponding STEM-EDX mapping images of (a-d) 2% Fe-W_2_C, (e-h) 2% Co-W_2_C, and (i-l) 2% Ni-W_2_C NSs. Figure S9: polarization curve of W substrate at the scan rate of 2 mV s^−1^ in 0.5 M H_2_SO_4_ solution. Figure S10: polarization curves of M-W_2_C electrodes (M=Ni, Co, and Fe) with varied (a) Ni, (b) Co, and (c) Fe contents. The content of M in the W_2_C lattice was determined by using ICP-OES elemental analysis. The measurements were conducted at the scan rate of 2 mV s^−1^ in 0.5 M H_2_SO_4_ solution (pH=0). Figure S11: Nyquist plots of W_2_C and 2% M-W_2_C (M=Fe, Co, and Ni) NSs. The EIS measurements were recorded at amplitude of 10 mV in 0.5 M H_2_SO_4_ solution. Inset: Randles circuit model, where R_s_ represents series resistance, C_dl_ represents double-layer capacitance, and R_ct_ represents the charge transfer resistance at the electrode-electrolyte interface. Figure S12: SEM images of 2% Ni-W_2_C NSs after chronoamperometry measurements for 28 h in (a) 0.5 M H_2_SO_4_ (pH = 0), (b) 0.1 M PBS (pH = 7.2), and (c) 1 M KOH (pH = 14). Figure S13: XRD patterns of 2% Ni-W_2_C NSs samples after chronoamperometry measurements for 28 h in (a) 0.5 M H_2_SO_4_ (pH = 0), (b) 1 M PBS (pH = 7.2), and (c) 1 M KOH (pH = 14). Figure S14: Ni 2p XPS spectrums of 2% Ni-W_2_C NSs after chronoamperometry measurements for 28 h in (a) 0.5 M H_2_SO_4_ (pH = 0), (b) 1 M PBS (pH=7.2), and (c) 1 M KOH (pH = 14). Figure S15: Faradaic efficiency of hydrogen generation measured within 300 min on 2% Ni-W_2_C NSs at current density of 80 mA cm^−2^ in (a, b) 0.5 M H_2_SO_4_ (pH = 0), (c, d) 1 M PBS (pH = 7.2), and (e, f) 1 M KOH (pH = 14). Table S1: composition analysis of the M-W_2_C (M= Fe, Co, and Ni) by ICP-OES and XPS. Table S2: onset overpotentials, operating overpotentials at current density j=10 mA cm^−2^, Tafel slopes, and exchange current densities of different samples obtained in 0.5 M H_2_SO_4_ solution. The amount of metal dopant was kept at 2* at*% for all the M-W_2_C NSs. Table S3: HER performances of the 2% Ni-W_2_C electrocatalyst in this study in comparison to various types of phosphide electrocatalysts in the literature. Table S4: HER performances of the 2% Ni-W_2_C electrocatalyst in this study in comparison to various types of sulfide electrocatalysts in the literature. Table S5: HER performances of the 2% Ni-W_2_C electrocatalyst in this study in comparison to various types of carbide electrocatalysts in the literature. Table S6: HER performances of the 2% Ni-W_2_C electrocatalyst in this study in comparison to various types of tungsten-based electrocatalysts in the literature. Table S7: HER performances of the 2% Ni-W_2_C electrocatalyst in this study in comparison to various types of the reported electrocatalysts used in the neutral electrolyte. Table S8: free energies of hydrogen adsorption to 3 are the possible adsorption sites (T, H1, and H2) on W_2_C nanosheet at low coverage,* i.e.*, the top of W atom (T), two trigonal sites with superimposing with C (H1), and bottom W atoms (H2).
