# Supplementary material for: Highly Efficient and Stable Hydrogen Production in All pH Range by Two-Dimensional Structured Metal-Doped Tungsten Semicarbides
Source: Research (Wash D C). 2019 May 2;2019:4029516. doi: 10.34133/2019/4029516 (PMC6750117; doi:10.34133/2019/4029516)
Supplement: Supplementary Materials — Scheme 1: schematic diagram illustrating the growth process of M-W2C NSs (M=Fe, Co, and Ni) on W substrate. Step 1: growth of WO3 NSs on the W substrate. Step 2: growth of the WO3/PVP/M on the presynthesized WO3 NSs. Step 3: formation of M-W2C NSs by carburizing the WO3/PVP/M NSs for HER. Figure S1: XRD pattern of WO3 NSs on W substrate. Figure S2: (a) XRD patterns, (b, c) magnified XRD patterns of W2C and W2C with various Fe (at%) doping contents, (d) magnified XRD patterns of W2C and W2C with various Fe (at%) doping contents using Cu as internal standard, (e, f) the plots of lattice parameters a and c versus Fe (at%) doping content measured by ICP-OES, and (g) the plot of unit cell volume of W2C versus the Fe (at%) doping content measured by ICP-OES. Figure S3: (a) XRD patterns, (b, c) magnified XRD patterns of W2C and W2C with various Co (at%) doping contents, (d) magnified XRD patterns of W2C and W2C with various Co (at%) doping contents using Cu as internal standard, (e, f) the plots of lattice parameters a and c versus Co (at%) doping content measured by ICP-OES, and (g) the plot of unit cell volume of W2C versus Co (at%) doping content measured by ICP-OES. Figure S4: schematic representation of the crystal structure of hexagonal W2C with a space group of P-3m1. Figure S5: SEM and TEM characterizations of (a, c) 2% Fe-W2C and (b, d) 2% Co-W2C NSs, (a, b) FESEM images, and (c, d) HRTEM images (insets: corresponding SAED patterns). Figure S6: SEM image of the cross-section view of pure W2C NSs on W substrate. Figure S7: AFM image of pure W2C NSs and the corresponding height profile along the white dashed line. Figure S8: HAADF images and their corresponding STEM-EDX mapping images of (a-d) 2% Fe-W2C, (e-h) 2% Co-W2C, and (i-l) 2% Ni-W2C NSs. Figure S9: polarization curve of W substrate at the scan rate of 2 mV s−1 in 0.5 M H2SO4 solution. Figure S10: polarization curves of M-W2C electrodes (M=Ni, Co, and Fe) with varied (a) Ni, (b) Co, and (c) Fe contents. The c [file 4029516.f1.docx]

Supplementary Materials


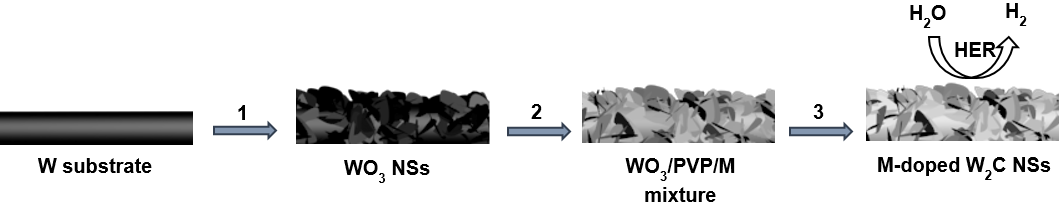


**Scheme 1.** Schematic diagram illustrating the growth process of M-W_2_C NSs (M=Fe, Co, Ni) on W substrate. Step 1: Growth of WO_3_ NSs on the W substrate. Step 2: Growth of the WO_3_/PVP/M on the pre-synthesized WO_3_ NSs. Step 3: Formation of M-W_2_C NSs by carburizing the WO_3_/PVP/M NSs for HER.


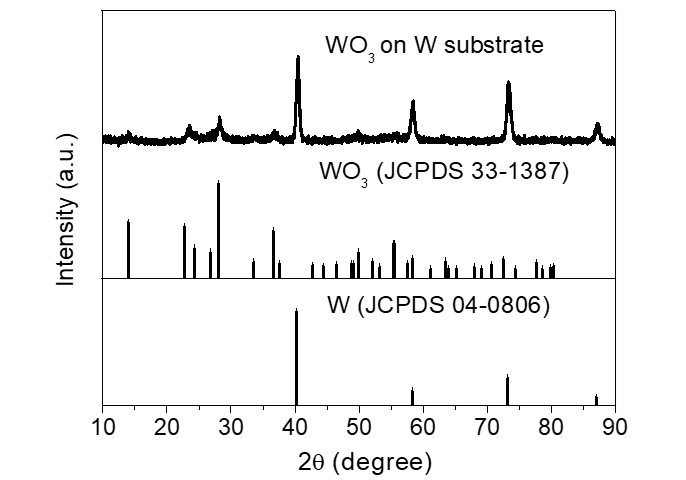


**Figure S1.** XRD pattern of WO_3_ NSs on W substrate.


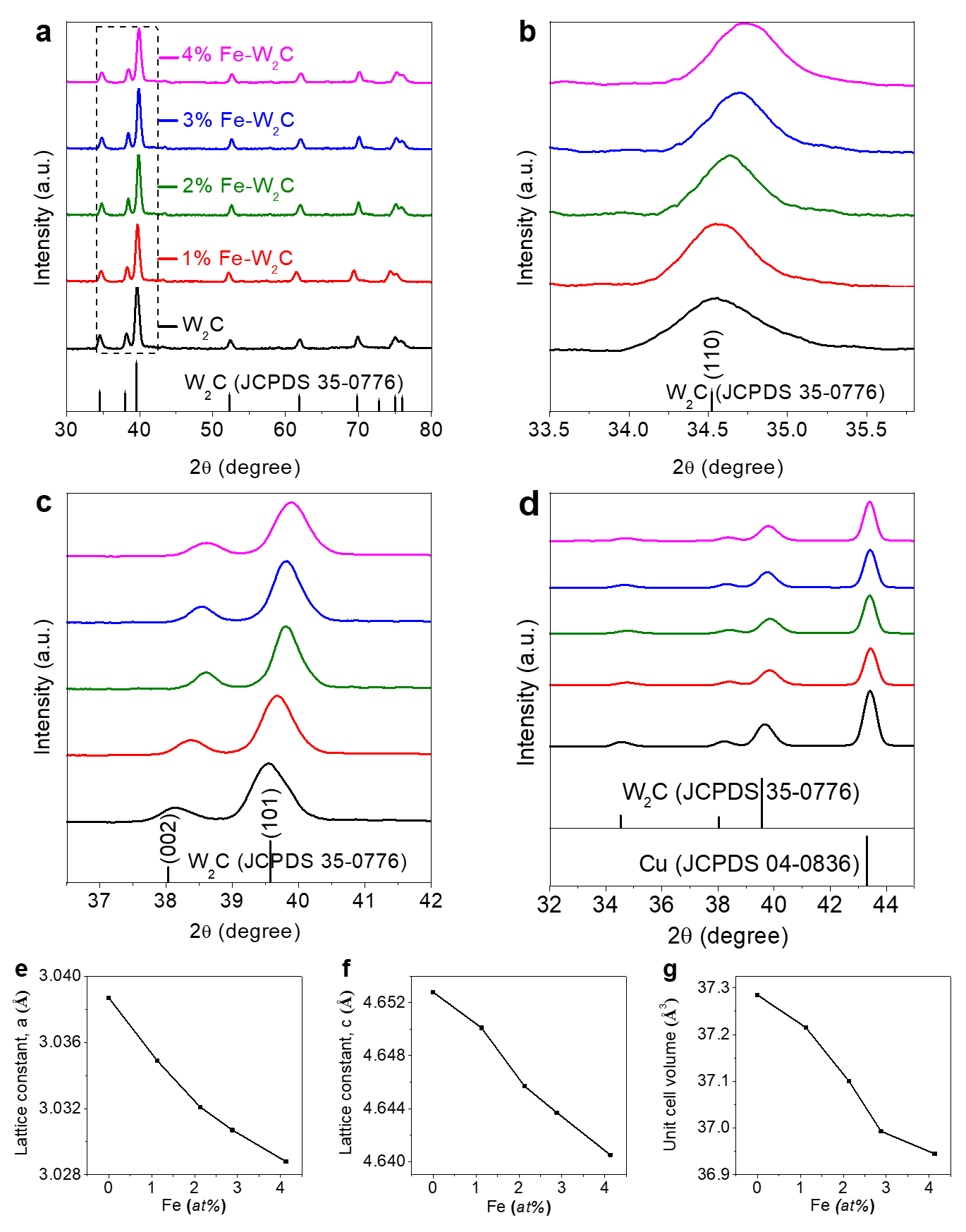


**Figure S2.** (a) XRD patterns and (b,c) magnified XRD patterns of W_2_C and W_2_C with various Fe (*at%*) doping contents. (d) Magnified XRD patterns of W_2_C and W_2_C with various Fe (*at%*) doping contents using Cu as internal standard. (e,f) The plots of lattice parameters a and c versus Fe (*at%*) doping content measured by ICP-OES. (g) The plot of unit cell volume of W_2_C versus the Fe (*at%*) doping content measured by ICP-OES.

After doping with Fe, all the XRD peaks of (110), (002), and (101) showed right shift with the increase of Fe doping amount (Figure S2a-d). The Cu was used as the internal reference, which did not show a detectable peak shift in the XRD measurements. It indicates that the peak shift observed in the Fe-W_2_C came from the doping of Fe into W_2_C. Using the Rietveld refinement method on the XRD pattern, the lattice parameters and the unit cell volume were calculated, which clearly showed that the lattice constant of a and c of W_2_C decreased after the Fe doping (Figure S2e,f), resulting in the decrease of the unit cell volume (Figure S2g).


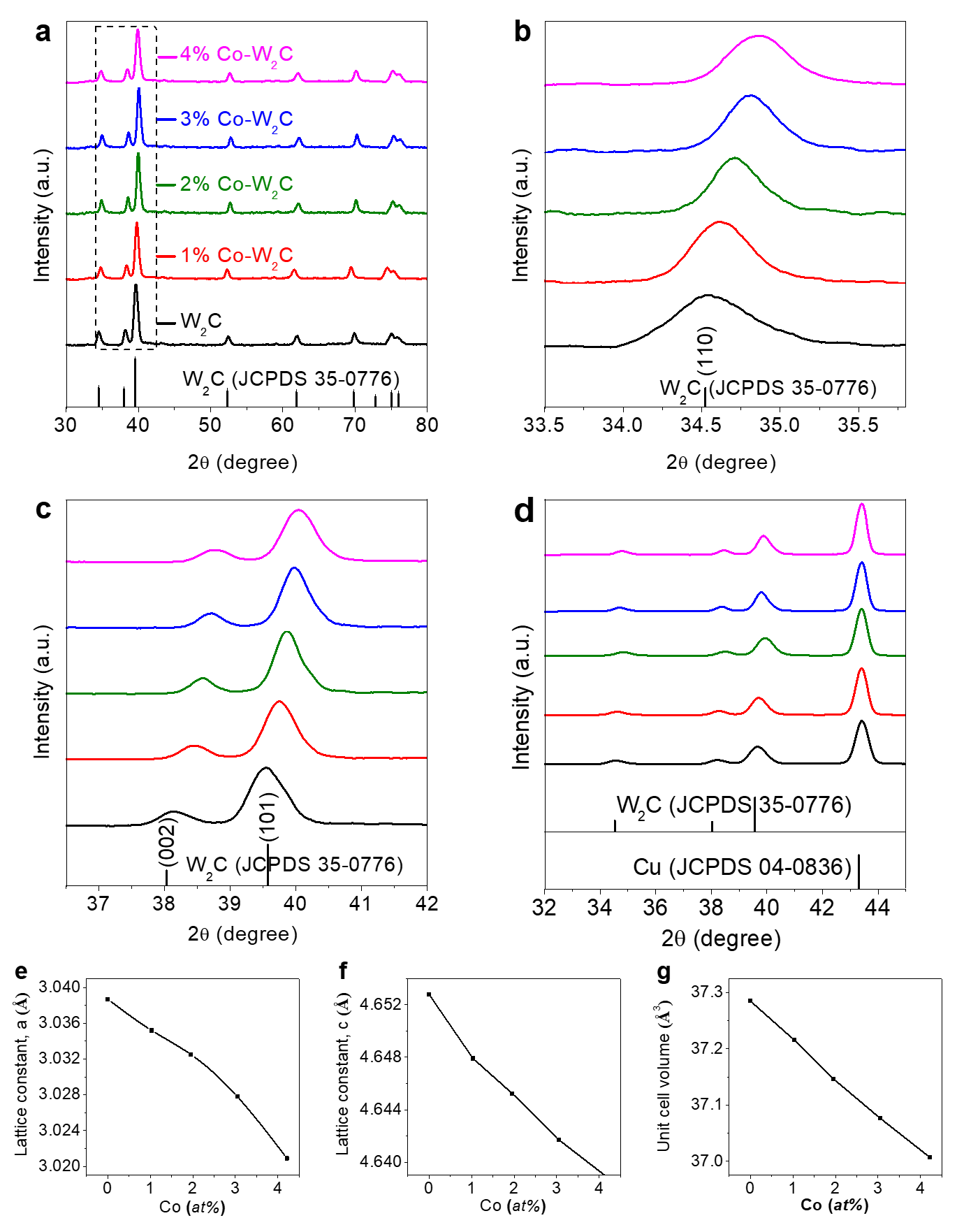


**Figure S3.** (a) XRD patterns and (b,c) magnified XRD patterns of W_2_C and W_2_C with various Co (*at%*) doping contents. (d) Magnified XRD patterns of W_2_C and W_2_C with various Co (*at%*) doping contents using Cu as internal standard. (e,f) The plots of lattice parameters a and c versus Co (*at%*) doping content measured by ICP-OES. (g) The plot of unit cell volume of W_2_C versus Co (*at%*) doping content measured by ICP-OES.

After doping with Co, all the XRD peaks of (110), (002), and (101) showed right shift with the increase of Co doping amount (Figure S3a-d). The Cu was used as the internal reference, which did not show a detectable peak shift in the XRD measurements. It indicates that the peak shift observed in the Co-W_2_C came from the doping of Co into W_2_C. Using the Rietveld refinement method on the XRD pattern, the lattice parameters and the unit cell volume were calculated, which clearly showed that the lattice constant of a and c of W_2_C decreased after the Co doping (Figure S3e,f), resulting in the decrease of the unit cell volume (Figure S3g).


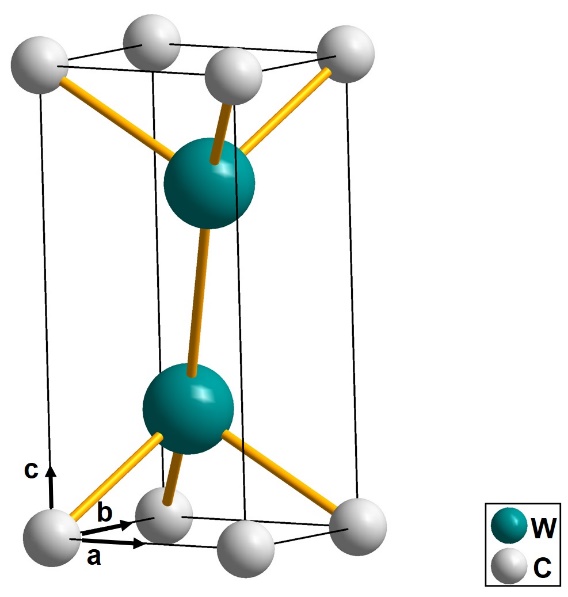


**Figure S4.** Schematic representation of the crystal structure of hexagonal W_2_C with a space group of P-3m1.


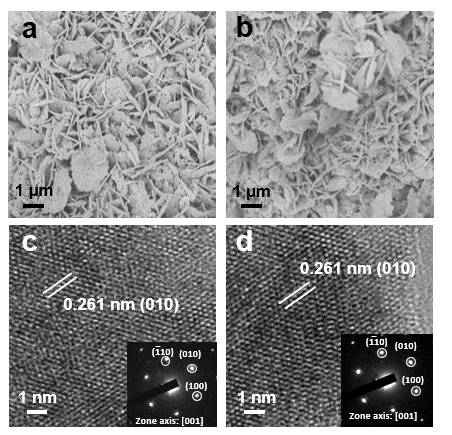


**Figure S5.** SEM and TEM characterizations of (a,c) 2% Fe-W_2_C and (b,d) 2% Co-W_2_C NSs. (a,b) FESEM images. (c,d) HRTEM images (Insets: corresponding SAED patterns).


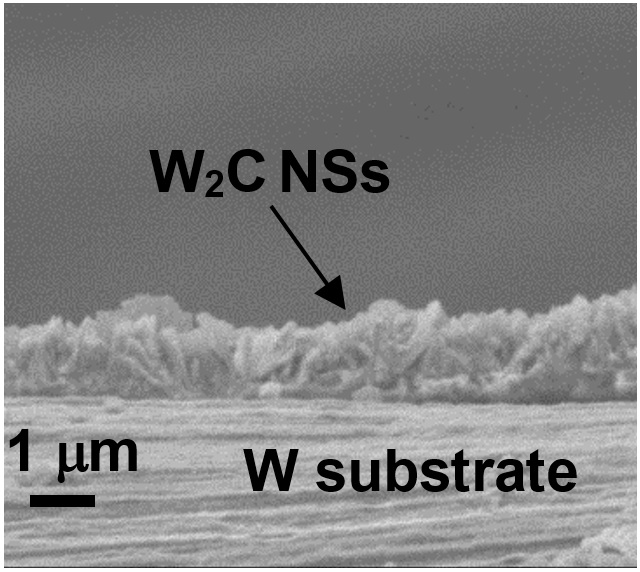


**Figure S6.** SEM image of the cross-section view of pure W_2_C NSs on W substrate.


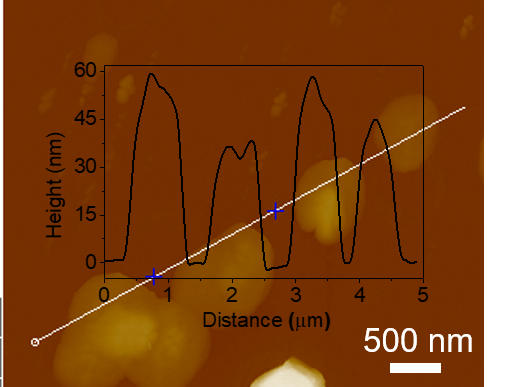


**Figure S7.** AFM image of pure W_2_C NSs and the corresponding height profile along the white dashed line.


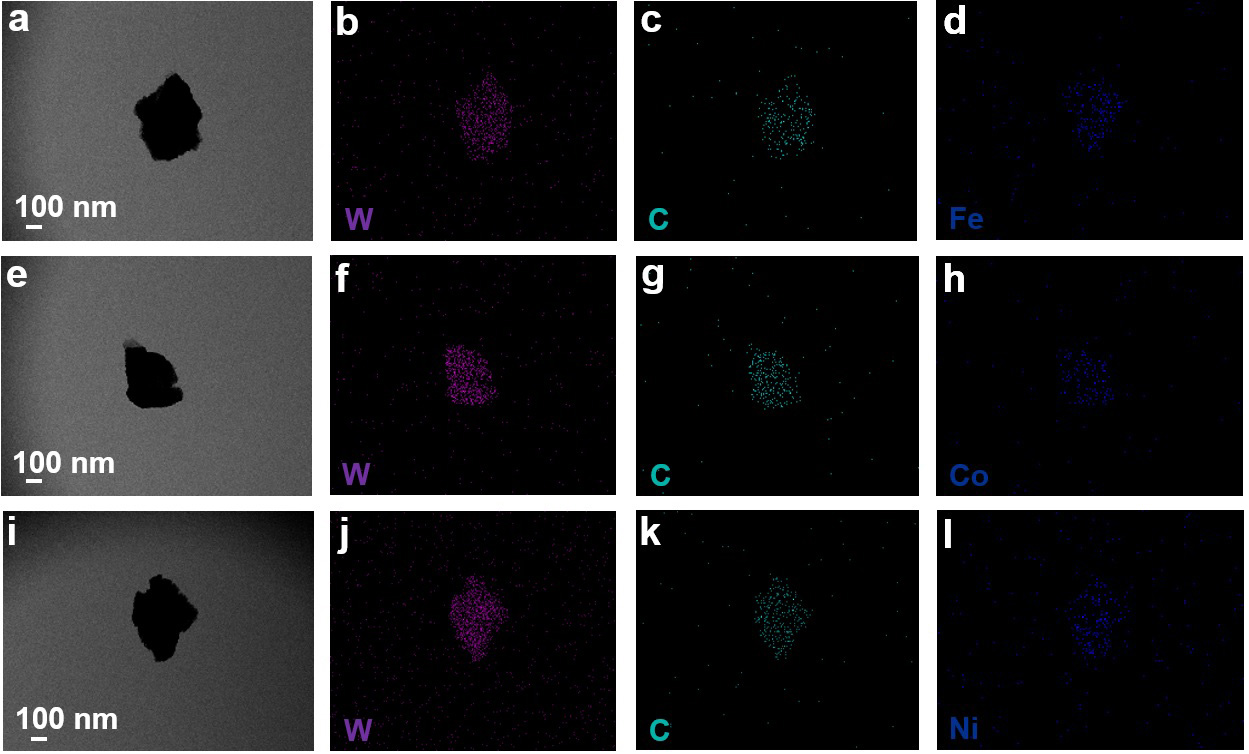


**Figure S8.** HAADF images and their corresponding STEM-EDX mapping images of (a-d) 2% Fe-W_2_C, (e-h) 2% Co-W_2_C, and (i-l) 2% Ni-W_2_C NSs.


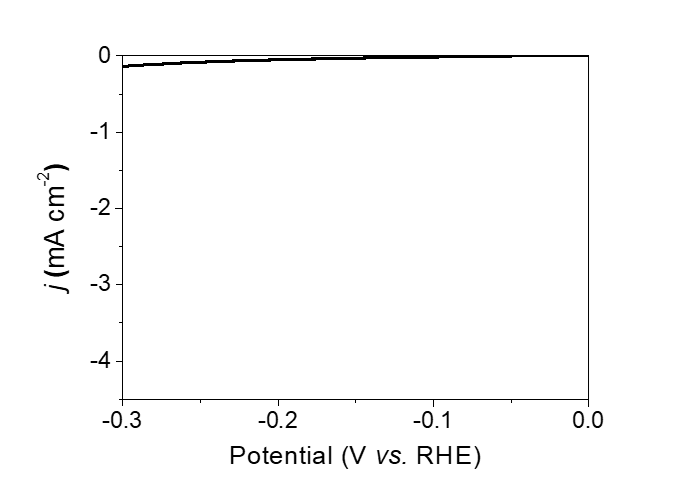


**Figure S9.** Polarization curve of W substrate at the scan rate of 2 mV s^-1^ in 0.5 M H_2_SO_4_ solution.


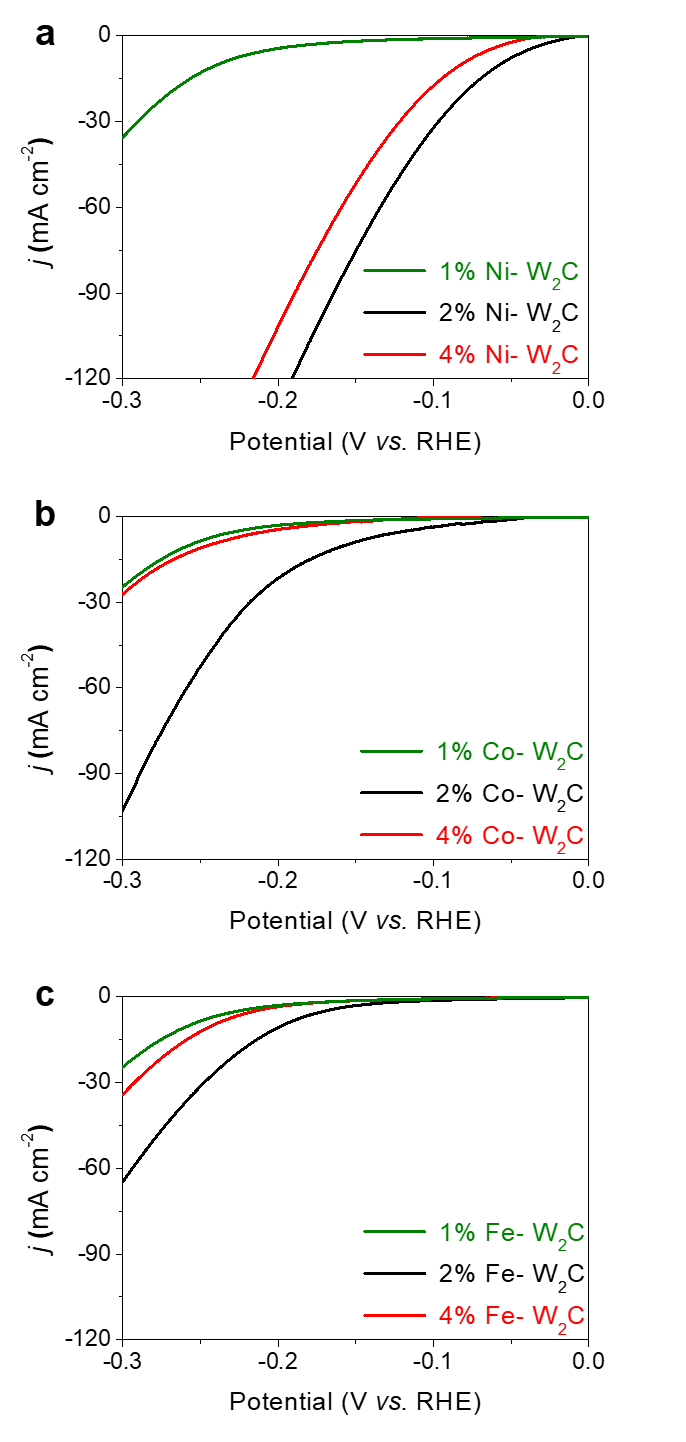


**Figure S10.** Polarization curves of M-W_2_C electrodes (M=Ni, Co, Fe) with varied (a) Ni, (b) Co, (c) Fe contents. The content of M in the W_2_C lattice was determined by using ICP-OES elemental analysis. The measurements were conducted at the scan rate of 2 mV s^-1^ in 0.5 M H_2_SO_4_ solution (pH=0).


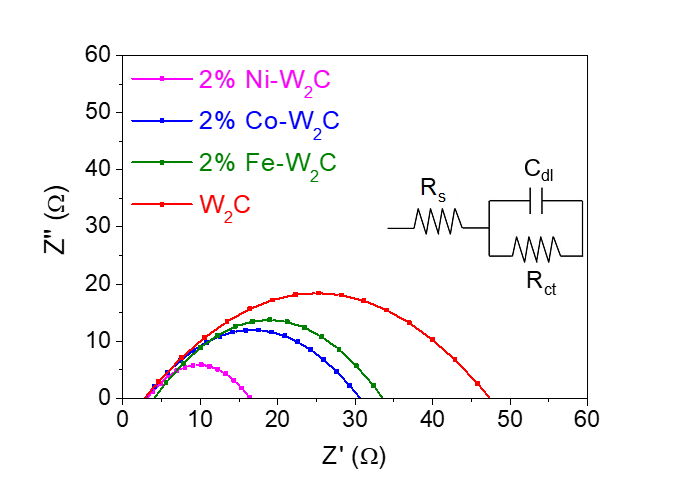


**Figure S11.** Nyquist plots of W_2_C and 2% M-W_2_C (M=Fe, Co, Ni) NSs. The EIS measurements were recorded at amplitude of 10 mV in 0.5 M H_2_SO_4_ solution. Inset: Randles circuit model, where R_s_ represents series resistance, C_dl_ represents double-layer capacitance, and R_ct_ represents the charge transfer resistance at the electrode-electrolyte interface.


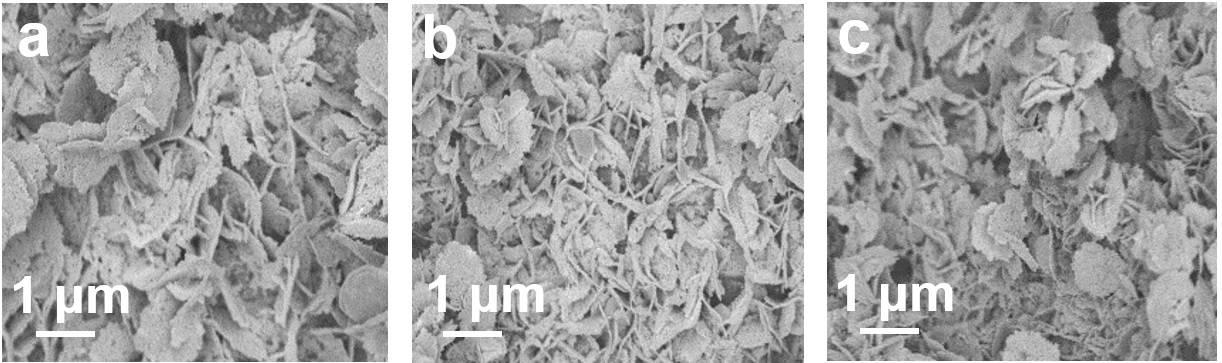


**Figure S12.** SEM images of 2% Ni-W_2_C NSs after chronoamperometry measurements for 28 h in (a) 0.5 M H_2_SO_4_ (pH = 0), (b) 0.1 M PBS (pH = 7.2), and (c) 1 M KOH (pH = 14).


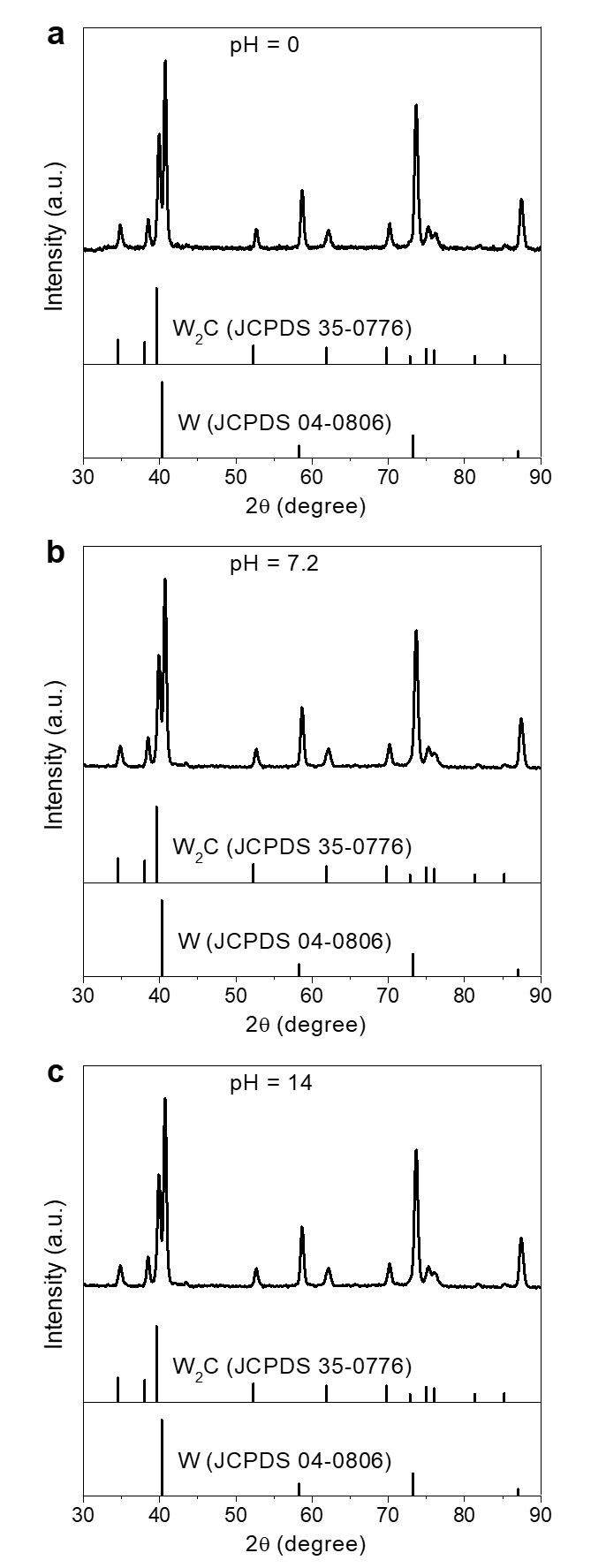


**Figure S13.** XRD patterns of 2% Ni-W_2_C NSs samples after chronoamperometry measurements for 28 h in (a) 0.5 M H_2_SO_4_ (pH = 0), (b) 1 M PBS (pH = 7.2), and (c) 1 M KOH (pH = 14).


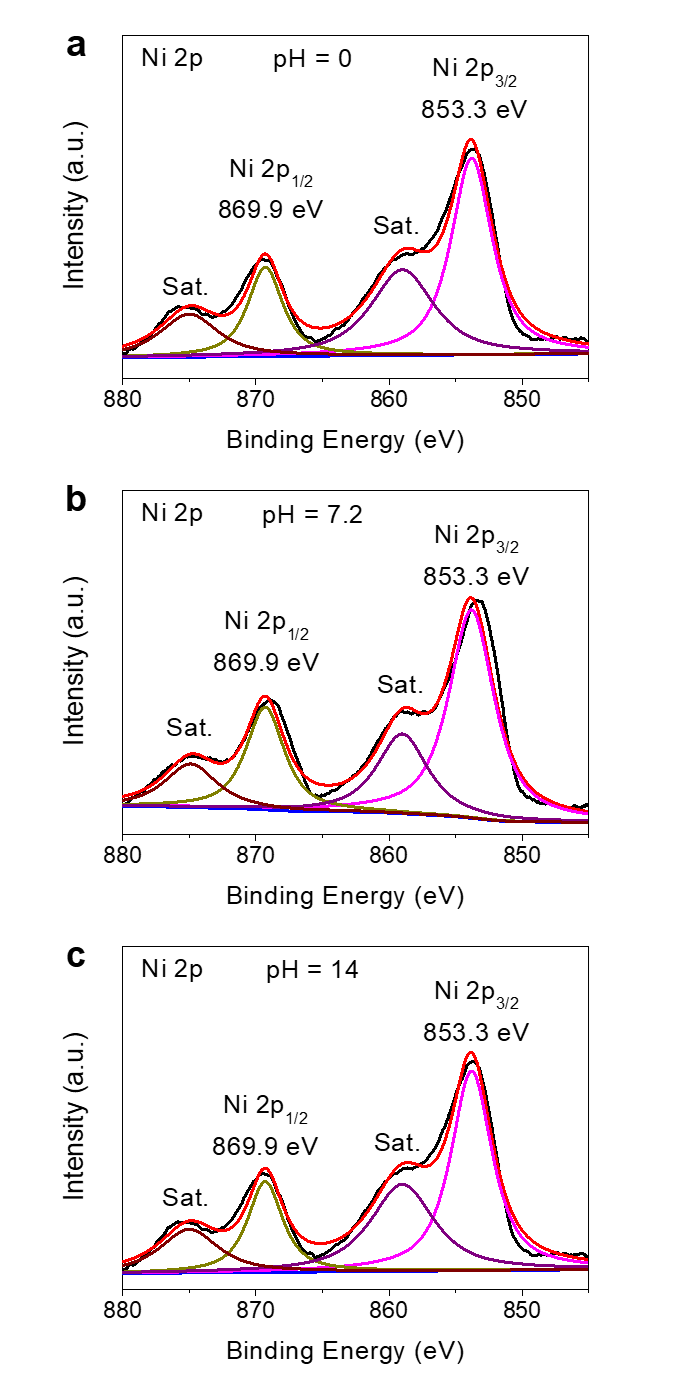


**Figure S14.** Ni 2p XPS spectrums of 2% Ni-W_2_C NSs after chronoamperometry measurements for 28 h in (a) 0.5 M H_2_SO_4_ (pH = 0), (b) 1 M PBS (pH=7.2), and (c) 1 M KOH (pH = 14).


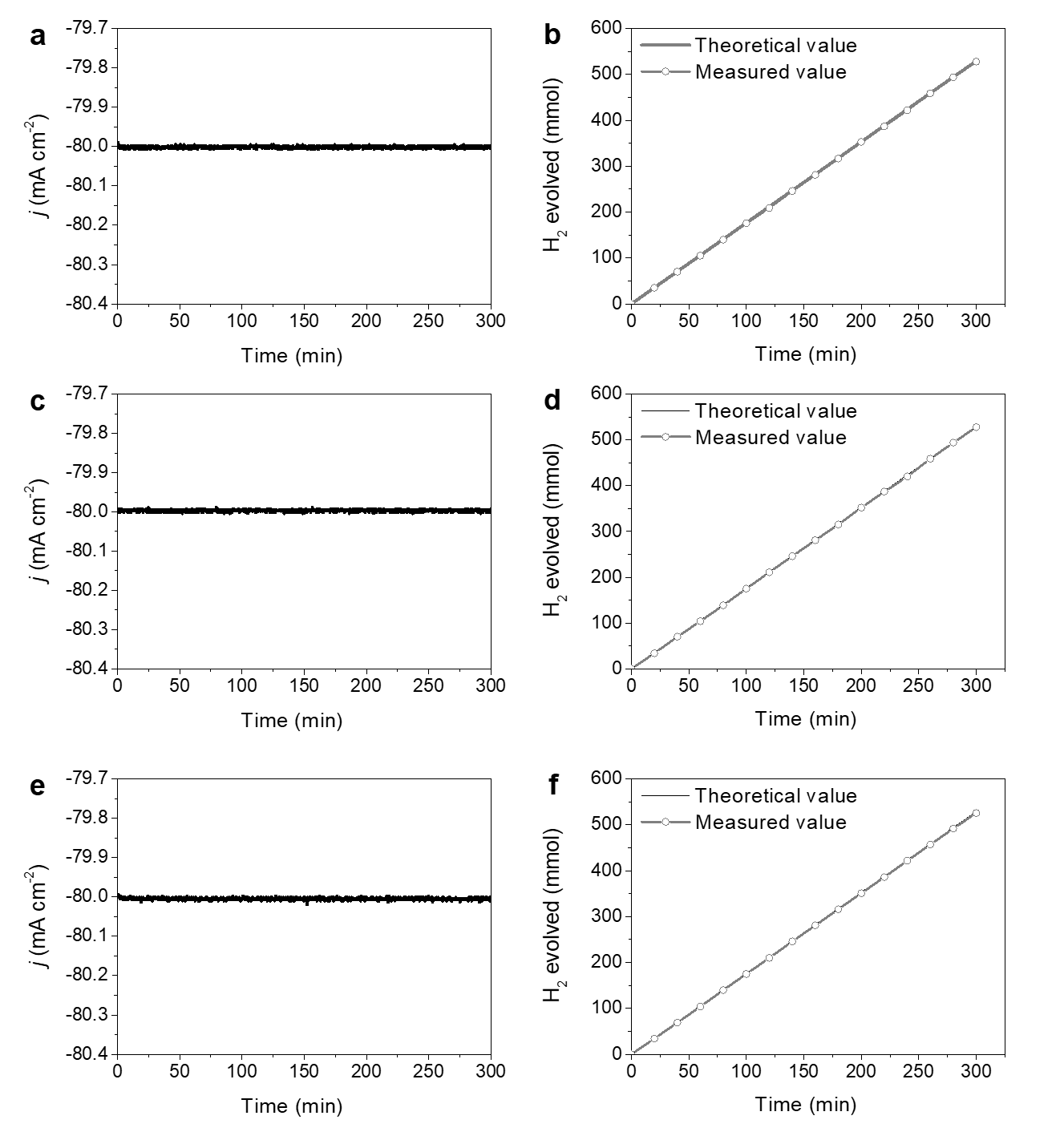


**Figure S15.** Faradaic efficiency of hydrogen generation measured within 300 min on 2% Ni-W_2_C NSs at current density of 80 mA cm^-2^ in (a,b) 0.5 M H_2_SO_4_ (pH = 0), (c,d) 1 M PBS (pH = 7.2), and (e,f) 1 M KOH (pH = 14).

**Table S1.** Composition analysis of the M-W_2_C (M= Fe, Co, Ni) by ICP-OES and XPS

| Samples | M content quantified  by ICP-OES | After HER in  0.5 M H_2_SO_4_ | After HER in  1.0 M PBS | After HER in  1.0 M KOH |
| --- | --- | --- | --- | --- |
| 1% Ni-W_2_C | 0.94 % | n.m | n.m | n.m |
| 2% Ni-W_2_C | 1.96 % | 1.93 %^*^ | 1.96 %^*^ | 1.88 %^*^ |
| 3% Ni-W_2_C | 2.89 % | n.m | n.m | n.m |
| 4% Ni-W_2_C | 3.95 % | n.m | n.m | n.m |
| 1% Co-W_2_C | 0.97 % | n.m | n.m | n.m |
| 2% Co-W_2_C | 1.93 % | n.m | n.m | n.m |
| 3% Co-W_2_C | 2.98 % | n.m | n.m | n.m |
| 4% Co-W_2_C | 3.86 % | n.m | n.m | n.m |
| 1% Fe-W_2_C | 0.97 % | n.m | n.m | n.m |
| 2% Fe-W_2_C | 1.92 % | n.m | n.m | n.m |
| 3% Fe-W_2_C | 2.92 % | n.m | n.m | n.m |
| 4% Fe-W_2_C | 3.88 % | n.m | n.m | n.m |

^*^quantified by XPS post analysis; n.m: not measured

**Table S2. Summary of electrochemical performances of the catalyst in this study.** Onset overpotentials, operating overpotentials at current density j=10 mA cm^-2^, Tafel slopes and exchange current densities of different samples obtained in 0.5 M H_2_SO_4_ solution. The amount of metal dopant was kept at 2 *at%* for all the M-W_2_C NSs.

| **Electrocatalyst** | **Onset overpotential (mV)** | **η_10_ (mV)** | **Tafel slope (mV dec^-1^)** | **j_o_ (mA cm^-2^)** |
| --- | --- | --- | --- | --- |
| W_2_C NSs | 122 | 274 | 145 | 0.19 |
| 2% Fe-W_2_C NSs | 78 | 197 | 102 | 0.22 |
| 2% Co-W_2_C NSs | 45 | 157 | 122 | 0.41 |
| 2% Ni-W_2_C NSs | 4 | 57 | 39 | 0.79 |
| Pt/C | 0 | 39 | 30 | 0.92 |

**Table S3.** HER performances of the 2% Ni-W_2_C electrocatalyst in this study in comparison to various types of phosphide electrocatalysts in the literature.

| **Electrocatalyst** | **Working electrode** | **Electrolyte** | **Onset overpotential (mV)** | **η_10_ (mV)** | **Tafel slope (mV dec^-1^)** | **j_0_**  **(mA cm^-2^)** | **Mass loading (mg cm^-2^)** | **Ref.** |
| --- | --- | --- | --- | --- | --- | --- | --- | --- |
| FeP NPs | Ti plate | 0.5 M H_2_SO_4_ (pH=0.3) | - | 50 | 37 | 0.43 | 1 | ^[44]^ |
|  |  | 1.0 M PBS (pH=6.5) | - | 102 | - | - |  |  |
| FeP NAs | Ti plate | 0.5 M H_2_SO_4_ (pH=0) | 16 | 55 | 38 | 0.44 | 3.2 | [45] |
| CoP NCs/CNT | GCE | 0.5 M H_2_SO_4_ (pH=0) | 40 | 122 | 54 | 0.13 | 0.285 | [46] |
| CoP NPs | Ti foil | 0.5 M H_2_SO_4_ (pH=0) | 20 | 74 | 50 | 0.14 | 2 | [47] |
| CoP NPs | GCE | 0.5 M H_2_SO_4_ (pH=0) | - | 88 | 48 | - | 0.9 | [48] |
|  |  | 1.0 KOH (pH=14) | - | 170 | 66 | - | 0.175 |  |
| CoP NWs | Carbon cloth | 0.5 M H_2_SO_4_ (pH=0) | 38 | 67 | 51 | 0.288 | 0.92 | [49] |
|  |  | 1.0 PBS (pH=7.4 | 45 | 106 | 93 | - |  |  |
|  |  | 1.0 KOH (pH=14) | 80 | 209 | 129 | - |  |  |
| Co_2_P NRs | Ti plate | 0.5 M H_2_SO_4_ (pH=0) | 70 | 134 | 51.7 | - | 1.0 | [14] |
|  |  | 1.0 KOH (pH=14) | - | 160 | - | - |  |  |
| Ni_2_P NPs | Ti plate | 0.5 M H_2_SO_4_ (pH=0) | - | 116 | 46 | 0.033 | 1.0 | [47] |
|  |  | 1.0 KOH (pH=14) | - | -160 | - | - |  |  |
| Ni_5_P_4_ | Ni foil | 0.5 M H_2_SO_4_ (pH=0) | - | 140 | 40 | - | - | [50] |
|  |  | 1.0 KOH (pH=14) |  | 150 | 53 | - | - |  |
| Ni_5_P_4_ | Ti foil | 1.0 M H_2_SO_4_ (pH=0.3) |  | 23 | 33 | - | - | [51] |
|  |  | 1.0 NaOH (pH=14) |  | 49 | 98 | - | - |  |
| Cu_3_P NWs | Cu foam | 0.5 M H_2_SO_4_ (pH=0) | 62 | 120 | 67 | 0.18 | 15.2 | [52] |
| Interconnected MoP NPs | GCE | 0.5 M H_2_SO_4_ (pH=0) | 40 | 125 | 54 | 0.086 | 0.36 | [53] |
| MoP | GCE | 0.5 M H_2_SO_4_ (pH=0) | - | 126 | 54 | 0.034 | 0.86 | [13] |
|  |  | 1.0 KOH (pH=14) | - | 125 | 48 | 0.046 |  |  |
| Mo-W-P NSs | Carbon cloth | 0.5 M H_2_SO_4_ (pH=0) | - | 80 | 52 | 0.288 | 4.0 | [54] |
| WP_2_ NRs | GCE | 0.5 M H_2_SO_4_ (pH=0.3) | 56 | 148 | 52 | 0.013 | - | [55] |
|  |  | 1.0 PBS (pH=7.4) |  | 298 | 79 |  |  |  |
|  |  | 1.0 KOH (pH=14) |  | 225 | 84 |  |  |  |
| **2% Ni-W_2_C NSs** | **W substrate** | **0.5 M H_2_SO_4_ (pH=0)** | **4** | **57** | **39** | **0.79** | **1** | **This work** |
|  |  | **1 M PBS (pH=7.2)** | **9** | **63** | **51** | **0.61** |  |  |
|  |  | **1 M KOH (pH=14)** | **19** | **81** | **87** | **0.57** |  |  |

**Table S4.** HER performances of the 2% Ni-W_2_C electrocatalyst in this study in comparison to various types of sulfide electrocatalysts in the literature.

| **Electrocatalyst** | **Working electrode** | **Electrolyte** | **Onset overpotential (mV)** | **η_10_ (mV)** | **Tafel slope (mV dec^-1^)** | **j_0_**  **(mA cm^-2^)** | **Mass loading (mg cm^-2^)** | **Ref.** |
| --- | --- | --- | --- | --- | --- | --- | --- | --- |
| Co_0.1_Fe_0.9_S_2_ | Ti foil | 0.5 M H_2_SO_4_ (pH=0) | 90 | 160 | 46 | - | 0.4 | [56] |
| NiS nanoframe | Ni foam | 1.0 KOH (pH=14) | - | 94 | 139 | - | 2.0 | [57] |
| Fe-Ni-S NSs | Ti plate | 0.5 M H_2_SO_4_ (pH=0) | - | 105 | 40 | 0.02 | 0.254 | [58] |
| Ni-Mo-S NSs | Carbon Cloth | 0.5 M H_2_SO_4_ (pH=0) | 14 | 154 | 48 | - | 0.52 | [59] |
|  |  | 0.5 M PBS (pH=6.9) | 132 | 200 | 85.3 | 0.0489 |  |  |
| CoMoS_3_ hollow prisms | GCE | 0.5 M H_2_SO_4_ (pH=0) | 75 | 171 | 56.9 | 0.011 | 0.5 | [60] |
| Co_9_S_8_@MoS_2_/CNF | GCE | 0.5 M H_2_SO_4_ (pH=0) | 64 | 190 | 110 | - | 0.212 | [61] |
| Zn_0.30_Co_2.70_S_4_ | GCE | 0.5 M H_2_SO_4_ (pH=0) | 35 | 80 | 47.5 | 0.15 | 0.285 | [62] |
|  |  | 0.1 M PBS (pH=7.2) | - | 90 | - | - |  |  |
|  |  | 1.0 KOH (pH=14) | - | 85 | - | - |  |  |
| VS_2_ (1T) NSs | GCE | 0.5 M H_2_SO_4_ (pH=0) | - | 68 | 34 | - | 0.01 | [63] |
| MoS_2_/CoSe_2_ hybrid | GCE | 0.5 M H_2_SO_4_ (pH=0) | 50 | 68 | 36 | 0.073 | 0.28 | [16] |
| MoS_2_/rGO | GCE | 0.5 M H_2_SO_4_ (pH=0) | 140 | 160 | 41 | - | 0.28 | [64] |
| Double-gyroid MoS_2_ film | FTO | 0.5 M H_2_SO_4_ (pH=0) | 140 | 230 | 50 | 0.00069 | 0.06 | [61] |
| As-grown MoS_2_ (2H) | GCE | 0.5 M H_2_SO_4_ (pH=0) | - | ~300 | 110 | - | - | [65] |
| Li-intercalated MoS_2_ (1T) | GCE | 0.5 M H_2_SO_4_ (pH=0) | - | 187 | 43 | - | - |  |
| H_2_ treated MoS_2_ | GCE | 0.5 M H_2_SO_4_ (pH=0) | 300 | - | 147 | - | - | [66] |
| WS_2_ nanoflakes | GCE | 0.5 M H_2_SO_4_ (pH=0) | 100 | - | 48 | - | 0.35 | [67] |
| As-grown WS_2_ (2H) | GCE | 0.5 M H_2_SO_4_ (pH=0) | 200 | - | 110 | - | 0.0001-0.0065 | [68] |
| Li-intercalated WS_2_ (1T) | GCE | 0.5 M H_2_SO_4_ (pH=0) | 80 to 100 | - | 60 | - |  |  |
| As-grown WS_2_ (2H) | GCE | 0.5 M H_2_SO_4_ (pH=0) | - |  | 85 | - | 0.2-1.2 | [69] |
| Li-intercalated WS_2_ (1T) | GCE | 0.5 M H_2_SO_4_ (pH=0) | 75 |  | 70 | - |  |  |
| WS_2_@P,N,O-graphene | GCE | 0.5 M H_2_SO_4_ (pH=0) | - | 125 | 52.7 | 0.131 | 0.113 | [70] |
| **2% Ni-W_2_C NSs** | **W substrate** | **0.5 M H_2_SO_4_ (pH=0)** | **4** | **57** | **39** | **0.79** | **1** | **This work** |
|  |  | **1 M PBS (pH=7.2)** | **9** | **63** | **51** | **0.61** |  |  |
|  |  | **1 M KOH (pH=14)** | **19** | **81** | **87** | **0.57** |  |  |

**Table S5.** HER performances of the 2% Ni-W_2_C electrocatalyst in this study in comparison to various types of carbide electrocatalysts in the literature.

| **Electrocatalyst** | **Working electrode** | **Electrolyte** | **Onset overpotential (mV)** | **η_10_ (mV)** | **Tafel slope (mV dec^-1^)** | **j_0_**  **(mA cm^-2^)** | **Mass loading (mg cm^-2^)** | **Ref.** |
| --- | --- | --- | --- | --- | --- | --- | --- | --- |
| Fe_3_C/GNRs | GCE | 0.5 M H_2_SO_4_ (pH=0) | 32 | 49 | 46 | - | - | [38] |
| Co_3_C/GNRs |  | 0.5 M H_2_SO_4_ (pH=0) | 41 | 91 | 57 | - | - |  |
| Ni_3_C/GNRs |  | 0.5 M H_2_SO_4_ (pH=0) | 35 | 48 | 54 | - | - |  |
| Mo_2_C@NC(nitrogen carbon) | GCE | 0.5 M H_2_SO_4_ (pH=0) | - | 124 | 60 | - | 0.28 | [71] |
|  |  | 0.1 M PBS (pH=7.2) | - | 156 | - | - |  |  |
|  |  | 1.0 KOH (pH=14) | - | 60 | - | - |  |  |
| MoC_x_ nano-octahedrons | GCE | 0.5 M H_2_SO_4_ (pH=0) | 25 | 142 | 53 | 0.023 | 0.8 | [72] |
|  |  | 1.0 KOH (pH=14) | 80 | 151 | 59 | 0.029 |  |  |
| Mo_2_C NPs in carbon matrix | GCE | 0.5 M H_2_SO_4_ (pH=0) | 6 | 78 | 41 | 0.178 | 0.25 | [73] |
| Mo_2_C/CNT-GR | GCE | 0.5 M H_2_SO_4_ (pH=0) | 70 | 130 | 58 | 0.062 | 0.65 | [74] |
| β-Mo_2_C NPs | GCE | 0.1 M HClO_4_ (pH=1) | 50 | - | 120 | 0.0173 | 0.28 | [75] |
| β-Mo_2_C nanotubes | GCE | 0.5 M H_2_SO_4_ (pH=0) | 82 | 172 | 62 | 0.017 | 0.75 | [76] |
|  |  | 0.1 KOH (pH=13) | 37 | 112 | 55 | 0.087 |  |  |
| Mo_2_C NWs | GCE | 0.5 M H_2_SO_4_ (pH=0) | 70 | 130 | 53 | - | 0.21 | [77] |
| Mo_2_C@NPC(nitrogen, phosphrous codoped carbon shell/N, P codoped rGO) | GCE | 0.5 M H_2_SO_4_ (pH=0) | 0 | 34 | 33.6 | 1.09 | 0.14 | [78] |
| Mo_0.06_W_0.94_C/carbon black | GCE | 0.5 M H_2_SO_4_ (pH=0) | 156 | 220 | - | - | 0.7 | [79] |
| WC-1050 | GCE | 0.5 M H_2_SO_4_ (pH=0) | 15 | 145 | 72 | - | - | [22] |
|  |  | 0.1 KOH (pH=13) | 16 | 137 | 106 | - | - |  |
| **2% Ni-W_2_C NSs** | **W substrate** | **0.5 M H_2_SO_4_ (pH=0)** | **4** | **57** | **39** | **0.79** | **1** | **This work** |
|  |  | **1 M PBS (pH=7.2)** | **9** | **63** | **51** | **0.61** |  |  |
|  |  | **1 M KOH (pH=14)** | **19** | **81** | **87** | **0.57** |  |  |

**Table S6.** HER performances of the 2% Ni-W_2_C electrocatalyst in this study in comparison to various types of tungsten-based electrocatalysts in the literature.

| **Electrocatalyst** | **Working electrode** | **Electrolyte** | **Onset overpotential (mV)** | **η_10_ (mV)** | **Tafel slope (mV dec^-1^)** | **j_0_**  **(mA cm^-2^)** | **Mass loading (mg cm^-2^)** | **Ref.** |
| --- | --- | --- | --- | --- | --- | --- | --- | --- |
| Mo-W-P NSs | Carbon cloth | 0.5 M H_2_SO_4_ (pH=0) | - | 80 | 52 | 0.288 | 4.0 | [54] |
| WP_2_ NRs | GCE | 0.5 M H_2_SO_4_ (pH=0.3) | 56 | 148 | 52 | 0.013 | - | [55] |
|  |  | 1.0 PBS (pH=7.4) |  | 298 | 79 |  | - |  |
|  |  | 1.0 KOH (pH=14) |  | 225 | 84 |  | - |  |
| WS_2_ nanoflakes | GCE | 0.5 M H_2_SO_4_ (pH=0) | 100 | - | 48 | - | 0.35 | [67] |
| Li-intercalated WS_2_ (1T) | GCE | 0.5 M H_2_SO_4_ (pH=0) | 80 to -100 | - | 60 | - | 0.0001-0.0065 | [68] |
| Li-intercalated WS_2_ (1T) | GCE | 0.5 M H_2_SO_4_ (pH=0) | 75 |  | 70 | - | 0.8-1.2 | [69] |
| WS_2_@P,N,O-graphene | GCE | 0.5 M H_2_SO_4_ (pH=0) | - | 125 | 52.7 | 0.131 | 0.113 | [70] |
| WS_2(1-x)_Se_2x_ nanotube | Carbon fibers | 0.5 M H_2_SO_4_ (pH=0) | - | 260 | 105 | - | 0.21 | [80] |
| WC-1050 | GCE | 0.5 M H_2_SO_4_ (pH=0) | 15 | 145 | 72 | - | - | [22] |
|  | GCE | 0.1 KOH (pH=13) | 16 | 137 | 106 | - | - |  |
| WO_2_/carbon NWs | GCE | 0.5 M H_2_SO_4_ (pH=0) | 35 | 58 | 46 | 0.64 | 0.35 | [81] |
| WO_2.9_ | GCE | 0.5 M H_2_SO_4_ (pH=0) | - | 70 | 50 | 0.40 | 0.285 | [82] |
| P-WN/rGO | GCE | 0.5 M H_2_SO_4_ (pH=0) | 46 | 85 | 54 | 0.035 | 0.337 | [83] |
| Fe-WCN | GCE | pH=1 | - | 220 | 52 | - | 0.4 | [84] |
|  | GCE | pH=13 | - | 250 | - | - |  |  |
| **2% Ni-W_2_C NSs** | **W substrate** | **0.5 M H_2_SO_4_ (pH=0)** | **4** | **57** | **39** | **0.79** | **1** | **This work** |
|  |  | **1 M PBS (pH=7.2)** | **9** | **63** | **51** | **0.61** |  |  |
|  |  | **1 M KOH (pH=14)** | **19** | **81** | **87** | **0.57** |  |  |

**Table S7.** HER performances of the 2% Ni-W_2_C electrocatalyst in this study in comparison to various types of the reported electrocatalysts used in the neutral electrolyte.

| **Electrocatalyst** | **Working electrode** | **Electrolyte** | **Onset overpotentials (mV)** | **η_10_ (mV)** | **Tafel slope (mV dec^-1^)** | **j_0_**  **(mA cm^-2^)** | **Mass loading (mg cm^-2^)** | **Ref.** |
| --- | --- | --- | --- | --- | --- | --- | --- | --- |
| FeP NPs | Ti plate | 1.0 M PBS (pH=6.5) | - | 102 | - | - | 1.0 | [44] |
| CoP NWs | Carbon cloth | 1.0 PBS (pH=7.4) | 45 | 106 | 93 | - | 0.92 | [49] |
| WP_2_ NRs | GCE | 1.0 M PBS (pH=7.4) | - | 298 | 79 | - | - | [55] |
| Ni-Mo-S NSs | Carbon Cloth | 0.5 M NaPBS (pH=6.9) | 132 | 200 | 85.3 | 0.0489 | 0.52 | [59] |
| Zn_0.30_Co_2.70_S_4_ | GCE | 0.1 M PBS (pH=7.2) | - | 90 | - | - | 0.285 | [62] |
| Co-S | FTO | 1.0 M PBS (pH=7) | 43 | 160 | 93 | 0.256 | - | [85] |
| Cu_2_MoS_4_ | FTO | 0.1 M PBS (pH=7) | 135 | 337 | 95 | 0.04 | 0.0416 | [86] |
| Mo_2_C@NC(nitrogen carbon) | GCE | 0.1 M PBS (pH=7.2) | - | 156 | - | - | 0.28 | [71] |
| Co-NRCNT(nitrogen-rich CNT) | GCE | 0.1 M PBS (pH=7) | - | 540 | - | - | 0.28 | [87] |
| H_2_-Co catalyst | FTO | 0.5 M PBS (pH=7) | 290 | - | 140 | - | - | [88] |
| WC (grain size 16.5 nm) | GCE | 0.1 M NaPBS (pH=7) | - | >300 | - | - | - | [89] |
| **2% Ni-W_2_C NSs** | **W substrate** | **1 M PBS (pH=7.2)** | **9** | **63** | **51** | **0.61** | **1** | **This work** |

**Table S8.** Free energy of hydrogen adsorption to 3 are the possible adsorption sites (T, H1, and H2) on W_2_C nanosheet at low coverage, *i.e.* the top of an W atom (T), two trigonal sites with superimposing with C (H1) and bottom W atoms (H2).

| T | 0.223 eV |
| --- | --- |
| H1 | -0.635 eV |
| H2 | -0.707 eV |

References

44. J. F. Callejas, J. M. McEnaney, C. G. Read, J. C. Crompton, A. J. Biacchi, E. J. Popczun, T. R. Gordon, N. S. Lewis and R. E. Schaak "Electrocatalytic and Photocatalytic Hydrogen Production from Acidic and Neutral-pH Aqueous Solutions Using Iron Phosphide Nanoparticles," *ACS Nano*, vol. 8, pp. 11101-11107, 2014.

45. P. Jiang, Q. Liu, Y. Liang, J. Tian, A. M. Asiri and X. Sun "A Cost-Effective 3D Hydrogen Evolution Cathode with High Catalytic Activity: FeP Nanowire Array as the Active Phase," *Angewandte Chemie*, vol. 126, pp. 13069-13073, 2014.

46. Q. Liu, J. Tian, W. Cui, P. Jiang, N. Cheng, A. M. Asiri and X. Sun "Carbon Nanotubes Decorated with CoP Nanocrystals: A Highly Active Non-Noble-Metal Nanohybrid Electrocatalyst for Hydrogen Evolution," *Angewandte Chemie International Edition*, vol. 53, pp. 6710-6714, 2014.

47. E. J. Popczun, C. G. Read, C. W. Roske, N. S. Lewis and R. E. Schaak "Highly Active Electrocatalysis of the Hydrogen Evolution Reaction by Cobalt Phosphide Nanoparticles," *Angewandte Chemie International Edition*, vol. 53, pp. 5427-5430, 2014.

48. D.-H. Ha, B. Han, M. Risch, L. Giordano, K. P. C. Yao, P. Karayaylali and Y. Shao-Horn "Activity and stability of cobalt phosphides for hydrogen evolution upon water splitting," *Nano Energy*, vol. 29, pp. 37-45, 2016.

49. J. Tian, Q. Liu, A. M. Asiri and X. Sun "Self-Supported Nanoporous Cobalt Phosphide Nanowire Arrays: An Efficient 3D Hydrogen-Evolving Cathode over the Wide Range of pH 0–14," *Journal of the American Chemical Society*, vol. 136, pp. 7587-7590, 2014.

50. M. Ledendecker, S. Krick Calderón, C. Papp, H.-P. Steinrück, M. Antonietti and M. Shalom "The Synthesis of Nanostructured Ni5P4 Films and their Use as a Non-Noble Bifunctional Electrocatalyst for Full Water Splitting," *Angewandte Chemie International Edition*, vol. 54, pp. 12361-12365, 2015.

51. A. B. Laursen, K. R. Patraju, M. J. Whitaker, M. Retuerto, T. Sarkar, N. Yao, K. V. Ramanujachary, M. Greenblatt and G. C. Dismukes "Nanocrystalline Ni5P4: a hydrogen evolution electrocatalyst of exceptional efficiency in both alkaline and acidic media," *Energy & Environmental Science*, vol. 8, pp. 1027-1034, 2015.

52. J. Tian, Q. Liu, N. Cheng, A. M. Asiri and X. Sun "Self-Supported Cu3P Nanowire Arrays as an Integrated High-Performance Three-Dimensional Cathode for Generating Hydrogen from Water," *Angewandte Chemie*, vol. 126, pp. 9731-9735, 2014.

53. Z. Xing, Q. Liu, A. M. Asiri and X. Sun "Closely Interconnected Network of Molybdenum Phosphide Nanoparticles: A Highly Efficient Electrocatalyst for Generating Hydrogen from Water," *Advanced Materials*, vol. 26, pp. 5702-5707, 2014.

54. X.-D. Wang, Y.-F. Xu, H.-S. Rao, W.-J. Xu, H.-Y. Chen, W.-X. Zhang, D.-B. Kuang and C.-Y. Su "Novel porous molybdenum tungsten phosphide hybrid nanosheets on carbon cloth for efficient hydrogen evolution," *Energy & Environmental Science*, vol. 9, pp. 1468-1475, 2016.

55. H. Du, S. Gu, R. Liu and C. M. Li "Tungsten diphosphide nanorods as an efficient catalyst for electrochemical hydrogen evolution," *Journal of Power Sources*, vol. 278, pp. 540-545, 2015.

56. D.-Y. Wang, M. Gong, H.-L. Chou, C.-J. Pan, H.-A. Chen, Y. Wu, M.-C. Lin, M. Guan, J. Yang, C.-W. Chen, Y.-L. Wang, B.-J. Hwang, C.-C. Chen and H. Dai "Highly Active and Stable Hybrid Catalyst of Cobalt-Doped FeS2 Nanosheets–Carbon Nanotubes for Hydrogen Evolution Reaction," *Journal of the American Chemical Society*, vol. 137, pp. 1587-1592, 2015.

57. X.-Y. Yu, L. Yu, H. B. Wu and X. W. Lou "Formation of Nickel Sulfide Nanoframes from Metal–Organic Frameworks with Enhanced Pseudocapacitive and Electrocatalytic Properties," *Angewandte Chemie*, vol. 127, pp. 5421-5425, 2015.

58. X. Long, G. Li, Z. Wang, H. Zhu, T. Zhang, S. Xiao, W. Guo and S. Yang "Metallic Iron–Nickel Sulfide Ultrathin Nanosheets As a Highly Active Electrocatalyst for Hydrogen Evolution Reaction in Acidic Media," *Journal of the American Chemical Society*, vol. 137, pp. 11900-11903, 2015.

59. J. Miao, F.-X. Xiao, H. B. Yang, S. Y. Khoo, J. Chen, Z. Fan, Y.-Y. Hsu, H. M. Chen, H. Zhang and B. Liu "Hierarchical Ni-Mo-S nanosheets on carbon fiber cloth: A flexible electrode for efficient hydrogen generation in neutral electrolyte," *Science Advances*, vol. 1, pp. e1500259, 2015.

60. L. Yu, B. Y. Xia, X. Wang and X. W. Lou "General Formation of M–MoS3 (M = Co, Ni) Hollow Structures with Enhanced Electrocatalytic Activity for Hydrogen Evolution," *Advanced Materials*, vol. 28, pp. 92-97, 2016.

61. H. Zhu, J. Zhang, R. Yanzhang, M. Du, Q. Wang, G. Gao, J. Wu, G. Wu, M. Zhang, B. Liu, J. Yao and X. Zhang "When Cubic Cobalt Sulfide Meets Layered Molybdenum Disulfide: A Core–Shell System Toward Synergetic Electrocatalytic Water Splitting," *Advanced Materials*, vol. 27, pp. 4752-4759, 2015.

62. Z.-F. Huang, J. Song, K. Li, M. Tahir, Y.-T. Wang, L. Pan, L. Wang, X. Zhang and J.-J. Zou "Hollow Cobalt-Based Bimetallic Sulfide Polyhedra for Efficient All-pH-Value Electrochemical and Photocatalytic Hydrogen Evolution," *Journal of the American Chemical Society*, vol. 138, pp. 1359-1365, 2016.

63. J. Yuan, J. Wu, W. J. Hardy, P. Loya, M. Lou, Y. Yang, S. Najmaei, M. Jiang, F. Qin, K. Keyshar, H. Ji, W. Gao, J. Bao, J. Kono, D. Natelson, P. M. Ajayan and J. Lou "Facile Synthesis of Single Crystal Vanadium Disulfide Nanosheets by Chemical Vapor Deposition for Efficient Hydrogen Evolution Reaction," *Advanced Materials*, vol. 27, pp. 5605-5609, 2015.

64. Y. Li, H. Wang, L. Xie, Y. Liang, G. Hong and H. Dai "MoS2 Nanoparticles Grown on Graphene: An Advanced Catalyst for the Hydrogen Evolution Reaction," *Journal of the American Chemical Society*, vol. 133, pp. 7296-7299, 2011.

65. M. A. Lukowski, A. S. Daniel, F. Meng, A. Forticaux, L. Li and S. Jin "Enhanced Hydrogen Evolution Catalysis from Chemically Exfoliated Metallic MoS2 Nanosheets," *Journal of the American Chemical Society*, vol. 135, pp. 10274-10277, 2013.

66. G. Ye, Y. Gong, J. Lin, B. Li, Y. He, S. T. Pantelides, W. Zhou, R. Vajtai and P. M. Ajayan "Defects Engineered Monolayer MoS2 for Improved Hydrogen Evolution Reaction," *Nano Letters*, vol. 16, pp. 1097-1103, 2016.

67. L. Cheng, W. Huang, Q. Gong, C. Liu, Z. Liu, Y. Li and H. Dai "Ultrathin WS2 Nanoflakes as a High-Performance Electrocatalyst for the Hydrogen Evolution Reaction," *Angewandte Chemie International Edition*, vol. 53, pp. 7860-7863, 2014.

68. D. Voiry, H. Yamaguchi, J. Li, R. Silva, D. C. B. Alves, T. Fujita, M. Chen, T. Asefa, V. B. Shenoy, G. Eda and M. Chhowalla "Enhanced catalytic activity in strained chemically exfoliated WS2 nanosheets for hydrogen evolution," *Nature Materials*, vol. 12, pp. 850, 2013.

69. M. A. Lukowski, A. S. Daniel, C. R. English, F. Meng, A. Forticaux, R. J. Hamers and S. Jin "Highly active hydrogen evolution catalysis from metallic WS2 nanosheets," *Energy & Environmental Science*, vol. 7, pp. 2608-2613, 2014.

70. J. Duan, S. Chen, B. A. Chambers, G. G. Andersson and S. Z. Qiao "3D WS2 Nanolayers@Heteroatom-Doped Graphene Films as Hydrogen Evolution Catalyst Electrodes," *Advanced Materials*, vol. 27, pp. 4234-4241, 2015.

71. Y. Liu, G. Yu, G.-D. Li, Y. Sun, T. Asefa, W. Chen and X. Zou "Coupling Mo2C with Nitrogen-Rich Nanocarbon Leads to Efficient Hydrogen-Evolution Electrocatalytic Sites," *Angewandte Chemie*, vol. 127, pp. 10902-10907, 2015.

72. H. B. Wu, B. Y. Xia, L. Yu, X.-Y. Yu and X. W. Lou "Porous molybdenum carbide nano-octahedrons synthesized via confined carburization in metal-organic frameworks for efficient hydrogen production," *Nature Communications*, vol. 6, pp. 6512, 2015.

73. R. Ma, Y. Zhou, Y. Chen, P. Li, Q. Liu and J. Wang "Ultrafine Molybdenum Carbide Nanoparticles Composited with Carbon as a Highly Active Hydrogen-Evolution Electrocatalyst," *Angewandte Chemie*, vol. 127, pp. 14936-14940, 2015.

74. D. H. Youn, S. Han, J. Y. Kim, J. Y. Kim, H. Park, S. H. Choi and J. S. Lee "Highly Active and Stable Hydrogen Evolution Electrocatalysts Based on Molybdenum Compounds on Carbon Nanotube–Graphene Hybrid Support," *ACS Nano*, vol. 8, pp. 5164-5173, 2014.

75. C. Wan, Y. N. Regmi and B. M. Leonard "Multiple Phases of Molybdenum Carbide as Electrocatalysts for the Hydrogen Evolution Reaction," *Angewandte Chemie*, vol. 126, pp. 6525-6528, 2014.

76. F.-X. Ma, H. B. Wu, B. Y. Xia, C.-Y. Xu and X. W. Lou "Hierarchical β-Mo2C Nanotubes Organized by Ultrathin Nanosheets as a Highly Efficient Electrocatalyst for Hydrogen Production," *Angewandte Chemie*, vol. 127, pp. 15615-15619, 2015.

77. L. Liao, S. Wang, J. Xiao, X. Bian, Y. Zhang, M. D. Scanlon, X. Hu, Y. Tang, B. Liu and H. H. Girault "A nanoporous molybdenum carbide nanowire as an electrocatalyst for hydrogen evolution reaction," *Energy & Environmental Science*, vol. 7, pp. 387-392, 2014.

78. J.-S. Li, Y. Wang, C.-H. Liu, S.-L. Li, Y.-G. Wang, L.-Z. Dong, Z.-H. Dai, Y.-F. Li and Y.-Q. Lan "Coupled molybdenum carbide and reduced graphene oxide electrocatalysts for efficient hydrogen evolution," *Nature Communications*, vol. 7, pp. 11204, 2016.

79. S. T. Hunt, T. Nimmanwudipong and Y. Román-Leshkov "Engineering Non-sintered, Metal-Terminated Tungsten Carbide Nanoparticles for Catalysis," *Angewandte Chemie International Edition*, vol. 53, pp. 5131-5136, 2014.

80. K. Xu, F. Wang, Z. Wang, X. Zhan, Q. Wang, Z. Cheng, M. Safdar and J. He "Component-Controllable WS2(1–x)Se2x Nanotubes for Efficient Hydrogen Evolution Reaction," *ACS Nano*, vol. 8, pp. 8468-8476, 2014.

81. R. Wu, J. Zhang, Y. Shi, D. Liu and B. Zhang "Metallic WO2–Carbon Mesoporous Nanowires as Highly Efficient Electrocatalysts for Hydrogen Evolution Reaction," *Journal of the American Chemical Society*, vol. 137, pp. 6983-6986, 2015.

82. Y. H. Li, P. F. Liu, L. F. Pan, H. F. Wang, Z. Z. Yang, L. R. Zheng, P. Hu, H. J. Zhao, L. Gu and H. G. Yang "Local atomic structure modulations activate metal oxide as electrocatalyst for hydrogen evolution in acidic water," *Nature Communications*, vol. 6, pp. 8064, 2015.

83. H. Yan, C. Tian, L. Wang, A. Wu, M. Meng, L. Zhao and H. Fu "Phosphorus-Modified Tungsten Nitride/Reduced Graphene Oxide as a High-Performance, Non-Noble-Metal Electrocatalyst for the Hydrogen Evolution Reaction," *Angewandte Chemie International Edition*, vol. 54, pp. 6325-6329, 2015.

84. Y. Zhao, K. Kamiya, K. Hashimoto and S. Nakanishi "Hydrogen Evolution by Tungsten Carbonitride Nanoelectrocatalysts Synthesized by the Formation of a Tungsten Acid/Polymer Hybrid In Situ," *Angewandte Chemie International Edition*, vol. 52, pp. 13638-13641, 2013.

85. Y. Sun, C. Liu, D. C. Grauer, J. Yano, J. R. Long, P. Yang and C. J. Chang "Electrodeposited Cobalt-Sulfide Catalyst for Electrochemical and Photoelectrochemical Hydrogen Generation from Water," *Journal of the American Chemical Society*, vol. 135, pp. 17699-17702, 2013.

86. P. D. Tran, M. Nguyen, S. S. Pramana, A. Bhattacharjee, S. Y. Chiam, J. Fize, M. J. Field, V. Artero, L. H. Wong, J. Loo and J. Barber "Copper molybdenum sulfide: a new efficient electrocatalyst for hydrogen production from water," *Energy & Environmental Science*, vol. 5, pp. 8912-8916, 2012.

87. X. Zou, X. Huang, A. Goswami, R. Silva, B. R. Sathe, E. Mikmeková and T. Asefa "Cobalt-Embedded Nitrogen-Rich Carbon Nanotubes Efficiently Catalyze Hydrogen Evolution Reaction at All pH Values," *Angewandte Chemie International Edition*, vol. 53, pp. 4372-4376, 2014.

88. S. Cobo, J. Heidkamp, P.-A. Jacques, J. Fize, V. Fourmond, L. Guetaz, B. Jousselme, V. Ivanova, H. Dau, S. Palacin, M. Fontecave and V. Artero "A Janus cobalt-based catalytic material for electro-splitting of water," *Nature Materials*, vol. 11, pp. 802, 2012.

89. F. Harnisch, G. Sievers and U. Schröder "Tungsten carbide as electrocatalyst for the hydrogen evolution reaction in pH neutral electrolyte solutions," *Applied Catalysis B: Environmental*, vol. 89, pp. 455-458, 2009.
